# Exercise Mitigates Alcohol Induced Endoplasmic Reticulum Stress Mediated Cognitive Impairment through ATF6-Herp Signaling

**DOI:** 10.1038/s41598-018-23568-z

**Published:** 2018-03-26

**Authors:** Akash K. George, Jyotirmaya Behera, Kimberly E. Kelly, Nandan K. Mondal, Kennedy P. Richardson, Neetu Tyagi

**Affiliations:** Department of Physiology, University of Louisville School of medicine, Louisville, KY 40202 USA

## Abstract

Chronic ethanol/alcohol (AL) dosing causes an elevation in homocysteine (Hcy) levels, which leads to the condition known as Hyperhomocysteinemia (HHcy). HHcy enhances oxidative stress and blood-brain-barrier (BBB) disruption through modulation of endoplasmic reticulum (ER) stress; in part by epigenetic alternation, leading to cognitive impairment. Clinicians have recommended exercise as a therapy; however, its protective effect on cognitive functions has not been fully explored. The present study was designed to observe the protective effects of exercise (EX) against alcohol-induced epigenetic and molecular alterations leading to cerebrovascular dysfunction. Wild-type mice were subjected to AL administration (1.5 g/kg-bw) and subsequent treadmill EX for 12 weeks (5 day/week@7–11 m/min). AL affected mouse brain through increases in oxidative and ER stress markers, SAHH and DNMTs alternation, while decreases in CBS, CSE, MTHFR, tight-junction proteins and cellular H_2_S levels. Mechanistic study revealed that AL increased epigenetic DNA hypomethylation of Herp promoter. BBB dysfunction and cognitive impairment were observed in the AL treated mice. AL mediated transcriptional changes were abolished by administration of ER stress inhibitor DTT. In conclusion, exercise restored Hcy and H_2_S to basal levels while ameliorating AL-induced ER stress, diminishing BBB dysfunction and improving cognitive function via ATF6-Herp-signaling. EX showed its protective efficacy against AL-induced neurotoxicity.

## Introduction

Alcoholism is a serious public health concern worldwide. Alcohol misuse and abuse has severe deleterious effects on physical and mental health. Excessive chronic alcohol intake can lead to a wide range of neuropsychiatric or neurological disorders, cardiovascular disease, liver disease, cancers and so on^1–6^. Public health strategies to reduce alcohol intake have involved alcohol risk awareness programs and measures aimed at controlling its availability^7^. Despite these measures, alcohol intake and alcohol related problems remain high and thus there is a need for strategies aimed at minimizing the health risks associated with alcohol intake.

Continuous alcohol intake can lead to changes in the function and morphology of brain. Growing evidence suggests that alcoholism is associated with imbalance in the sulfur amino acid metabolism^8^. Homocysteine (Hcy) is a sulfur-containing non-proteinogenic amino acid formed as a by-product of methyl-transfer reactions in methionine metabolism^9^. Alcohol interferes with the Hcy metabolism in multiple ways that leads to increased accumulation of Hcy in plasma -condition known as hyperhomocysteinemia (HHcy)^10–12^. Hcy neurotoxicity via overstimulation of N-methyl-D-aspartate receptors may contribute to the pathogenesis of both brain shrinkage and withdrawal of seizures linked to alcoholism^13^. HHcy is linked to increased risk of vascular disease and vascular dementia (Lehmann *et al*.)^14^, Alzheimer’s disease (AD) and mild cognitive impairment (MCI)^15–17^. Studies also revealed that, Hcy levels predict cognitive decline in healthy adults^18–20^, and HHcy is an independent risk factor for the development of dementia^21^.

The endoplasmic reticulum (ER) is involved in regulation of posttranslational protein processing and transport. The accumulation of unfolded or misfolded proteins in the ER lumen triggers ER stress, which activates the so-called unfolded protein response (UPR)^22^. Extensive and acute ER stress direct the UPR towards activation of death-triggering pathways^23^. The expression of ER stress markers, such as ATF6 (activating transcription factor 6), GPR78 (glucose-regulated protein 78), PERK (Protein kinase RNA-like endoplasmic reticulum kinase), EIF2 (eukaryotic initiation factor 2), and IRE1 (Inositol-requiring enzyme 1) are altered in AD patient samples^24–26^. Enhanced ER stress further potentiates the production of reactive oxygen species (ROS) in multiple pathways^27^. An optimum level of ROS is essential for signaling and normal cell functioning. Excessive ROS is generated during alcohol metabolism or Hcy auto-oxidation, thus causing the disruption of redox homeostasis and affecting the redox signaling pathways^28^. Moreover, Hcy has been found to induce neurological dysfunction via oxidative stress through increased production of amyloid-β peptide^29^. However, administration of antioxidants like N-acetyl cysteine, vitamin C or E could prevent Hcy mediated cytotoxicity^29^. It is also reported that antioxidants (vitamin E or C) may have a preventive role during memory dysfunction caused by HHcy in rats^30^.

Generation of gaseous transmitters by mammalian tissue system has attracted much attention in past few years. The accumulating evidence suggests that, despite its past concept as a noxious gas, H_2_S (hydrogen sulfide) is rapidly emerging as a third gaseous transmitter, in addition to nitric oxide (NO) and carbon monoxide (CO)^31^ that regulates blood pressure and vasomotor activity, controls inflammation, and angiogenesis^32^. H_2_S was found to be produced endogenously in various parts of the body such as the heart, blood, and central nervous system by two pyridoxal-5′ -phosphate-dependent enzymes responsible for metabolism of l-cysteine which is a by-product of l-methionine, Hcy, and cystathione^33^. Two major sources for endogenous enzymatic production of H_2_S are cystathionine β synthase (CBS) and cystathionine γ lyase (CSE)^34^. Other reported that along with CBS and CSE, a newly identified enzyme, 3-mercaptopyruvate sulfur transferase (3MST) is also involved in generation of endogenous H_2_S^35^. CBS is the major H_2_S producing enzyme in the brain^36^. H_2_S can easily penetrate the plasma membrane thus inducing wide spectrum of signaling cascades in the target cells. Some studies in cellular and animal models have suggested several mechanisms to explain the protection associated with H_2_S, including promoting anti-inflammatory^37^, anti-apoptotic^38^, vasodilation and neuroprotection^39^. In addition, H_2_S and NO effects signaling pathways have been described to offer protection against Alzheimer’s amyloid vasculopathy and neurodegeneration^40^. There are reports of H_2_S induced endothelial proliferation and migration and enhanced VEGF (vascular endothelial growth factor) gene expression^41^. H_2_S also increases the production of intracellular GSH (glutathione), a major intracellular antioxidant and thus alleviating ER stress and promoting neuronal protection and improving brain function^42,43^.

Physical exercise has been accepted as a major strategy to improve public health. There is convincing evidence showing that regular exercise is associated with less mortality and greater recovery rates in various diseases^44–48^. Athletes with the highest training volume, exhibiting highest plasma folate levels, showed a decrease in Hcy levels followed by the training period^49,50^. Mechanistic research suggests that alcohol consumption and lack of exercise may be associated with chronic diseases. The recent work suggested that exercise may offset the increased risk for death associated with large amounts of alcoholconsumption^48^; although a detailed mechanism behind this observation is not available. In our current study, we showed that the alcohol induced oxidative stress mediated ER stress in the context of alcohol neurotoxicity and BBB (blood brain barrier) cognitive impairment and the beneficial role of exercise to rescue these phenotypes through endogenous H_2_S production.

## Results

### Effect of exercise on memory impairment during alcoholism in mice

**Figure 1:**
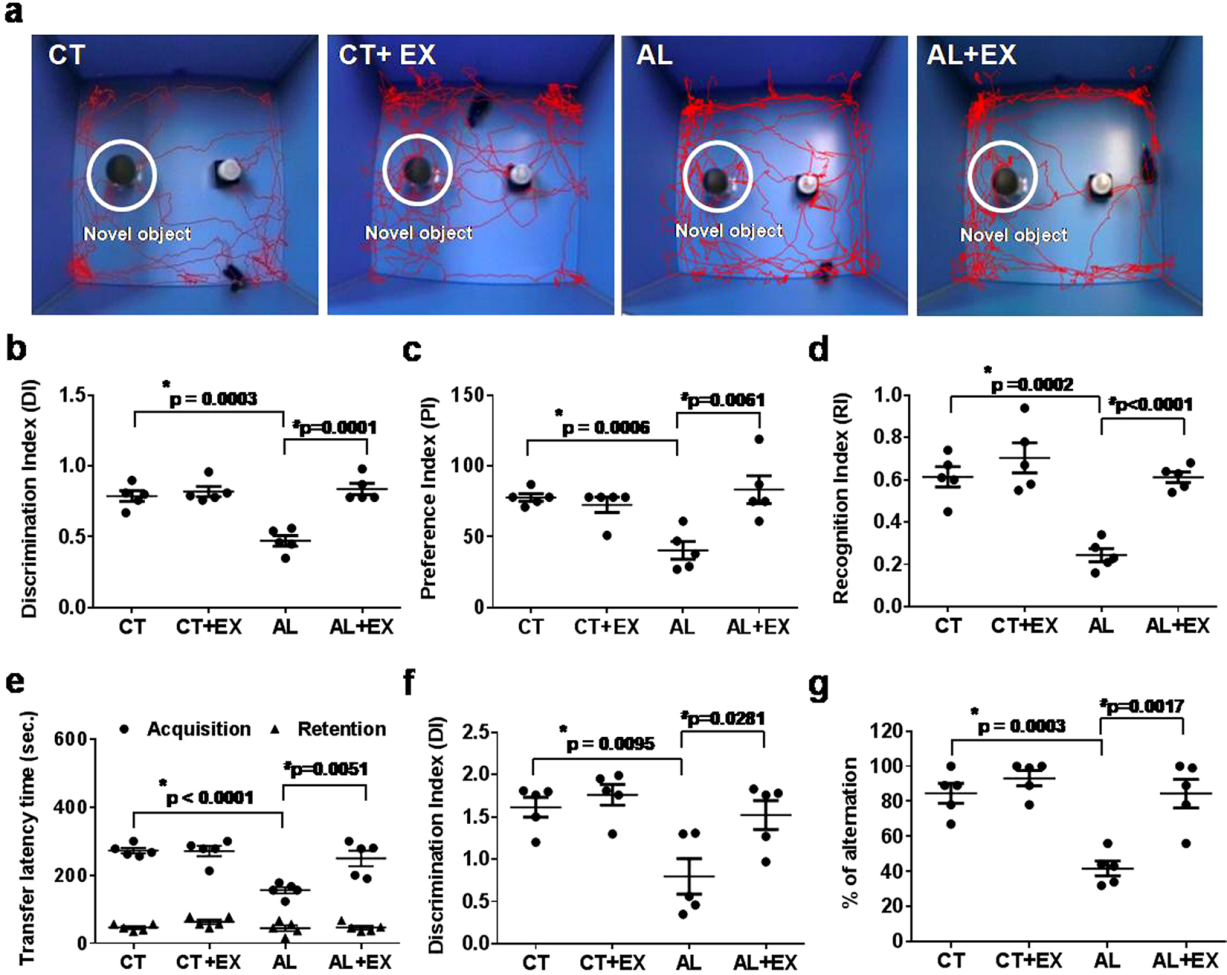
Effect of exercise on short-term and long-term memory and behavior under alcoholism. (**a–d**) Short-term memory of mice was assessed by the Novel Object Recognition Test (NORT). Movement patterns of experimental mice assessed by NORT (**a**). Values of discrimination index (**b**), preference index (**c**) and recognition index (**d**) for experimental groups are shown in scatter dot plots. (**e**) Similarly, long-term memory of mice was assessed by a Passive Avoidance Task (PAT). Transfer latency time data for the PAT analysis in experimental mice are shown in a scatter dot plot. (**f,g**) Y-maze data of discrimination index (**f**) and percentage of spontaneous alterations (**g**) for experimental groups are shown in scatter dot plots. Data are represented as mean values ± standard error (SE) in 5 independent experiments. *^,#^p < 0.05 considered significant. *p < 0.05 vs. CT and ^#^p < 0.05 vs. AL group.

The Novel Object Recognition Test (NORT), Passive Avoidance Task (PAT) and Y-maze test were performed to test memory function in mice. We tested experimental mice in performing a novel object recognition task that relies on the mouse’s natural exploratory behavior. Figure 1a displays the schematic representation of the experimental protocol, in which mice were habituated to the open-field apparatus for 3 consecutive days. The mice were then allowed to explore two identical objects. On the 3^rd^ day, one hour after the last exploration, mice were presented with a familiar and a new object. Mice in the alcohol (AL) treated group exhibited significantly impaired novel object recognition performance in the simple task relative to control (CT) mice in exploring the novel object. The discrimination indexes (DI), preference indexes (PI) and recognition indexes (RI) in AL treated mice were significantly reduced in comparison to the CT group (Fig. 1b–d). AL treated mice with regular exercise practice (AL+EX) exhibited improvement in preference between novel and familiar object recognition when compared to only AL treated mice. Similarly, we analyzed long term memory using the PAT, and our results showed that the AL induced long term memory dysfunction was improved by exercise in the AL+EX mice group (Fig. 1e). The Y-maze test results showed that there was improved memory and behavioral improvisation in the AL+EX group compared to the AL group (Fig. 1f,g). Similarly, the DI and percentage of alternation data derived from the Y-maze experiment showed that active exercise in AL treated mice improved their memory function when compared to the AL group without exercise (Fig. 1f,g).

### Effect of exercise on mean arterial blood pressure and cerebral blood flow

**Figure 2:**
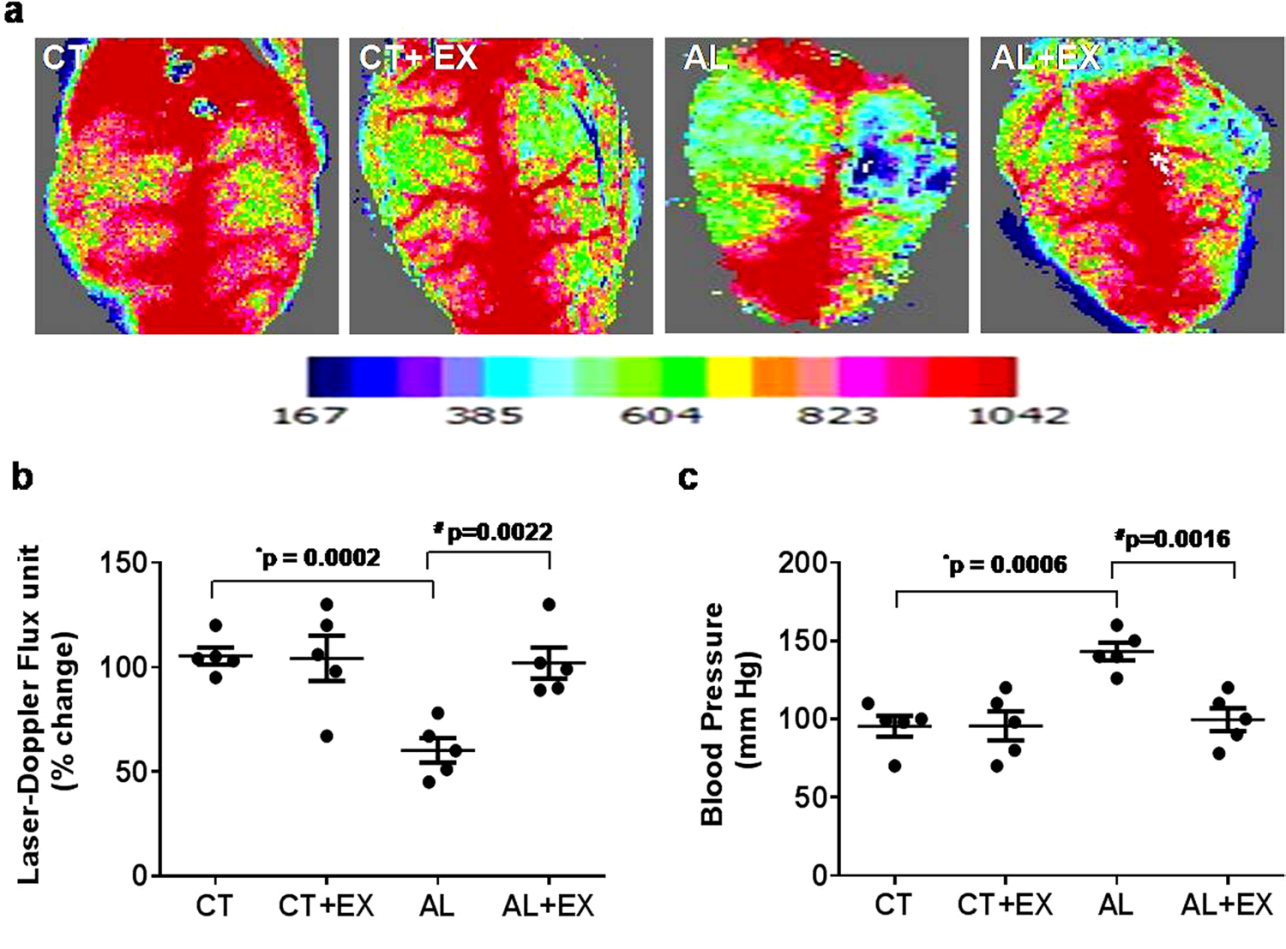
(**a**) Representative images showing microvascular density in the brain using laser Doppler flow in the different mice groups. (**b**) Scatter dot plot representing flux units of microvascular density captured in the 6 different areas in the cranial window prepared in the different groups of mice. (**c**) Dot plot of mean arterial blood pressure data in different mice groups. Data are represented as mean values ± standard error (SE) in 5 independent experiments. *^,#^p < 0.05 considered significant. *p < 0.05 vs. CT and ^#^p < 0.05 vs. AL group.

Cerebral Blood Flow (CBF) is necessary for conservation of brain function. In the current study, we measured alcohol-mediated cerebral blood flow. The results showed that AL treatment significantly decreased blood flow in the cerebral cortex as compared to control (CT) mice (Fig. 2a,b). Whereas, mean arterial blood pressure data displayed a significant increase in AL treated mice as compared to the CT group. Interestingly, the given treatment of exercise significantly alleviated blood flow as well as returned blood pressure to basal levels in AL+EX grouped mice (Fig. 2c), suggesting that exercise improves blood pressure and cerebral flow in experimental mice treated with alcohol.

### Alcohol administration interferes with Hcy metabolism leading to HHcy and exercise reversed this effect

**Figure 3:**
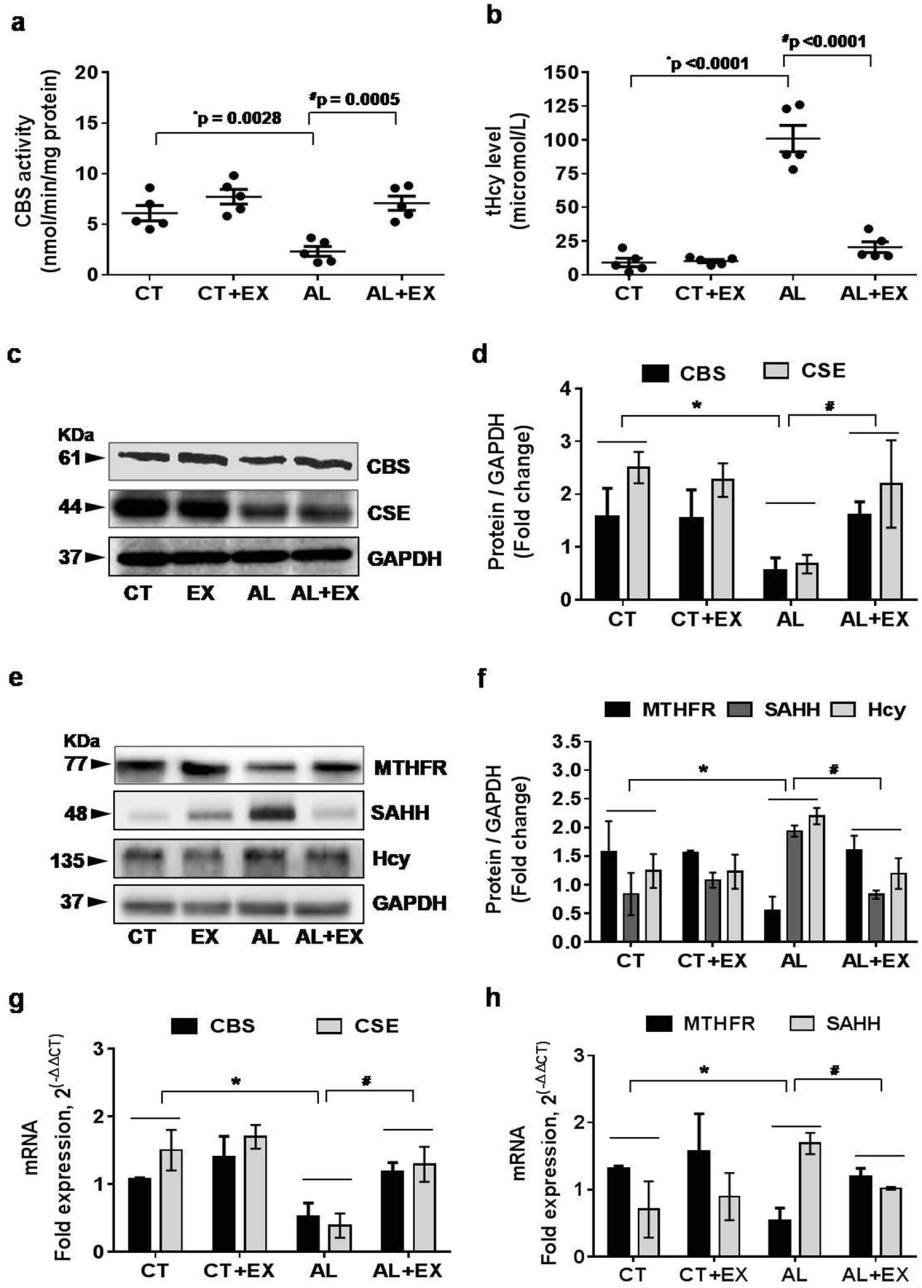
Alcohol interferes with Hcy metabolism leading to hyperhomocysteinemia (HHcy). (**a,b**) Scatter dot plots represent data for the CBS enzyme activity and total homocysteine (tHcy) levels in the different mice groups. (**c–f**) Representative western blot analysis for the vital enzymes (CBS, CSE, MTHFR, SAHH and Hcy) involved in homocysteine metabolism in different mice groups. Bar graphs showing quantitative estimation of key proteins after normalization with GAPDH. (**g,h**) q-PCR analysis showing the data for real‐time transcript levels of CBS, CSE, MTHFR and SAHH mRNAs in the different groups of mice. All the data are represented as mean values ± standard error (SE) in 5 independent experiments. *^,#^p < 0.05 considered significant. *p < 0.05 vs. CT and ^#^p < 0.05 vs. AL group.Uncropped blots for c and e are presented in Supplementary Figs 1 and 2.

To know the effect of exercise on the metabolic regulation of the alcohol-mediated Hcy pathway; we quantified the crucial enzymes involved in methionine metabolism to homocysteine, including CBS, CSE, methylene tetrahydrofolate reductase (MTHFR) and S-adenyl-L-homocysteine hydrolase (SAHH). Biochemical estimation of CBS enzyme activity data indicated that CBS enzyme activity was predominantly decreased in the alcoholic group with respect to the control group, while exercise treatment increased CBS activity in the AL+EX group as compared to AL treated mice (Fig. 3a). To evaluate the effect of exercise on the HHcy condition induced by alcohol, we analyzed the total plasma Hcy (tHcy) levels. The results suggested that the tHcy level was significantly increased in the alcoholic group with respect to the control group, but exercise restored the tHcy to the control level in the AL+EX group (Fig. 3b). Moreover, our western blot analysis of CBS, CSE, and Hcy in all experimental mice groups showed similar findings in agreement with biochemical estimations performed (Fig. 3c–f). Additionally, the q-PCR analysis of CBS and CSE mRNA expression confirmed our biochemical and protein data (Fig. 3g). Protein levels and mRNA expression of MTHFR were significantly reduced in the alcoholic group with respect to control; and they were reversed to normal levels in the in AL+EX group (Fig. 3e,f,h). Also, we observed significant up-regulation in protein and mRNA expression of SAHH in the alcoholic group compared to the control group. Interestingly, exercised treatment to the alcoholic group restored the expression of SAHH (Fig. 3e,f,h). Collectively, these results suggest that alcohol mediated altered expression of enzymes involved in the Hcy metabolism and exercise restored the altered levels of CBS, CSE, MTHFR and SAHH. These results suggest a protective role of exercise during alcoholism.

### Beneficial effect of exercise towards alcohol induced oxidative and ER stress

**Figure 4:**
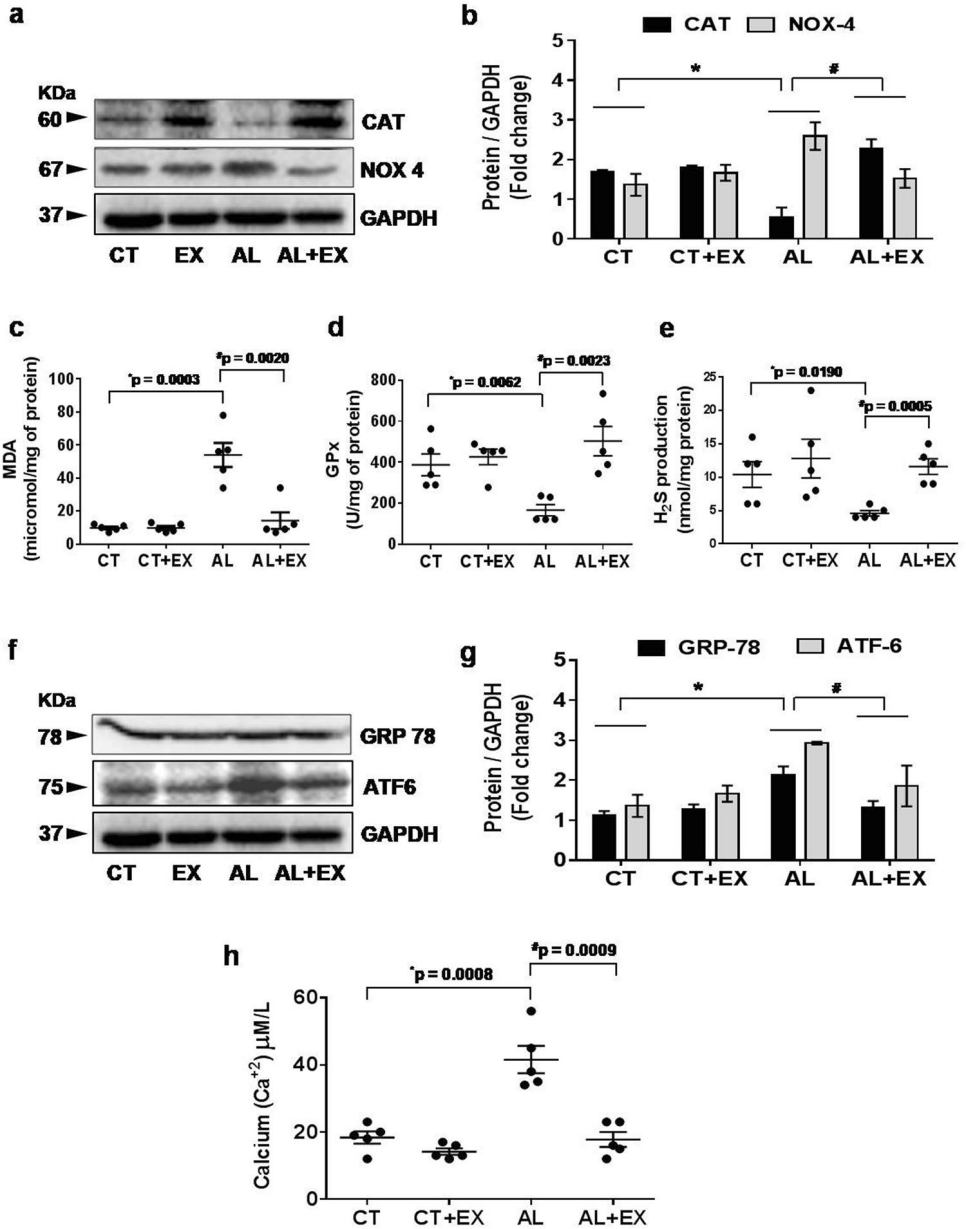
Effect of exercise on alcohol induced oxidative and endoplasmic reticular (ER) stress. (**a,b**) Representative western blot analysis showing the levels of antioxidant marker CAT and oxidative stress marker NOX4 in the different mice groups. Bar graphs showing the quantitative estimation of CAT and NOX4 proteins after normalization with GAPDH. (**c–e**) Scatter dot plots representing the levels of malondialdehyde (MDA), glutathione peroxidase (GPx) and production of H_2_S in brain tissue in different mice groups. (**f,g**) Representative western blot analysis showing the levels of GRP78 and ATF6 (hallmarks of ER stress) in the different groups of mice. Bar graphs showing the quantitative estimation of GRP78 and ATF6 proteins after normalization with GAPDH. (**h**) Scatter dot plot represents data for the cellular calcium ion (Ca^+2^) level in brain tissue extract of different mice groups. All the data are represented as mean values ± standard error (SE) in 5 independent experiments. *^,#^p < 0.05 considered significant. *p < 0.05 vs. CT and ^#^p < 0.05 vs. AL group. Uncropped blots for a and f are presented in Supplementary Fig. 3.

To evaluate the effect of alcohol on oxidative stress and the role of exercise in its mitigation, we performed western blot and biochemical analysis of different markers of oxidative stress in all experimental mice groups (Fig. 4a–e). Our data indicated that levels of NADPH oxidase 4 (NOX4) and malondialdehyde (MDA) were significantly upregulated in AL treated mice when compared to CT mice. On the other hand, the levels of catalase (CAT), glutathione peroxidase (GPx) and production of endogenous H_2_S were significantly reduced in the AL group compared to the CT group. These data indicated that there was an imbalance between oxidant and anti-oxidant status in the AL treated group, indicating a presence of robust oxidative stress levels. Exercise treatment proved to ameliorate oxidative stress in the AL+EX group.

To determine whether the increase in oxidative stress affected ER stress, protein expression analysis of GRP78 and ATF6 (hallmark proteins of ER stress) was performed. Interestingly, we observed that GRP78 and ATF6 expression levels were markedly increased in the AL treated group with respect to control. A reverse effect was observed in the exercise group. ER stress also caused calcium depletion. Therefore, we observed cellular calcium (Ca^+2^) levels in brain tissue extract. The results showed that alcohol increased the Ca^+2^ depletion and exercise reversed this alcohol mediated effect. These results demonstrate that hyperhomocysteinemia triggered not only oxidative stress but also triggered ER stress with respect to the reduction in Ca^+2^ levels.

### Protective role of exercise on alcohol induced epigenetic remodeling

**Figure 5:**
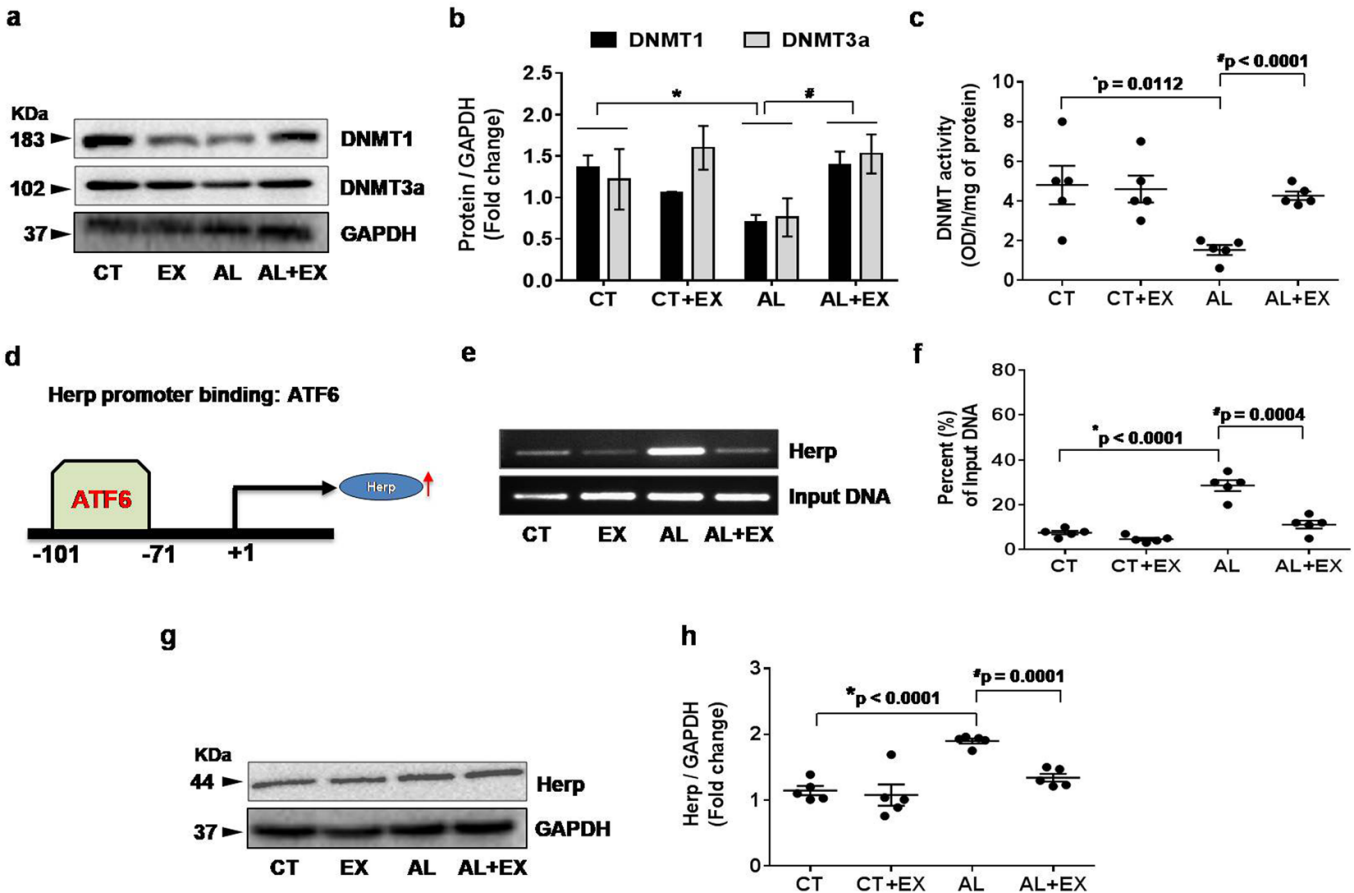
Effect of exercise on alcohol induced DNA methyltransferase (DNMT) activity and transcriptional regulation through ATF6-Herp signaling. (**a–c**) Representative western blot analysis showing the levels of DNMT1 and DNMT3a in brain tissue extract from the different mice groups. Bar graphs showing the quantitative estimation of DNMT1 and DNMT3a proteins after normalization with GAPDH. Scatter dot plot representing the DNMT activity in brain tissue of different mice groups. (**d–f**) ChIP assay to examine the ER activated ATF6 binding to the endogenous Herp promoter *in vivo*. After cross linking and immunoprecipitation with ATF6 antibody from isolated brain tissue extract, PCR was performed to identify the presence of Herp promoter DNA using primers flanking the CpG islands in the Herp promoter sites. Scatter dot plot representing the percent of input DNA in different experimental mice groups. (**g,h**) Representative western blot analysis showing the levels of Herp protein expression in brain tissue extract from the different mice groups. Scatter dot plot showing the quantitative estimation of Herp proteins after normalization with GAPDH. All the data are represented as mean values ± standard error (SE) in 5 independent experiments. *^,#^p < 0.05 considered significant. *p < 0.05 vs. CT and ^#^p < 0.05 vs. AL group. Uncropped blots for a,e,g are presented in Supplementary Figs 4 and 5.

To understand the effect of exercise on epigenetic changes, we measured the protein levels of DNA methyltransferase *1* (DNMT1) and DNA methyltransferase3 alpha (DNMT3a) by western blotting in the brain tissue extracts of experimental mice groups. The results revealed that, DNMT1 and DNMT3a protein levels were significantly decreased under alcoholism when compared to the control group. However, regular practice of exercise was able to reverse the alcohol mediated decreased DNMT expression (Fig. 5a,b). Moreover, biochemical estimation of DNMT activity in experimental mice confirmed our protein data (Fig. 5c).

### ATF6 transcriptionally activates cellular Herp during the alcohol dependent ER stress response and exercise mitigates this activation

To determine transcriptional regulation of homocysteine-induced endoplasmic reticulum protein (Herp) by direct binding of ATF6; we performed a ChIP (chromatin immunoprecipitation) assay to examine the ER activated ATF6 binding to endogenous Herp promoter *in vivo* (Fig. 5d–f). After crosslinking and immunoprecipitation with ATF6 antibody, PCR was performed to identify the presence of Herp DNA promoter using primers flanking the CpG islands in the Herp promoter sites. Our results demonstrate that ATF6 antibody readily precipitated chromatin containing the Herp promoter in the AL group with respect to CT. However, its effects are minimized under exercise. These results indicate that ATF6 binds to the Herp promoter and governs gene expression, specifically in, in *vivo* conditions under alcoholism. Since Herp is believed to be regulated under transcriptional regulation of ATF6 during ER stress^51^, we further performed western blot analysis to study Herp expression. The results indicate that alcohol strongly induces Herp expression and exercise lowered its expression down to control levels (Fig. 5g,h).

### Exercise improved the alcohol induced vascular permeability and blood brain barrier dysfunction in mice

**Figure 6:**
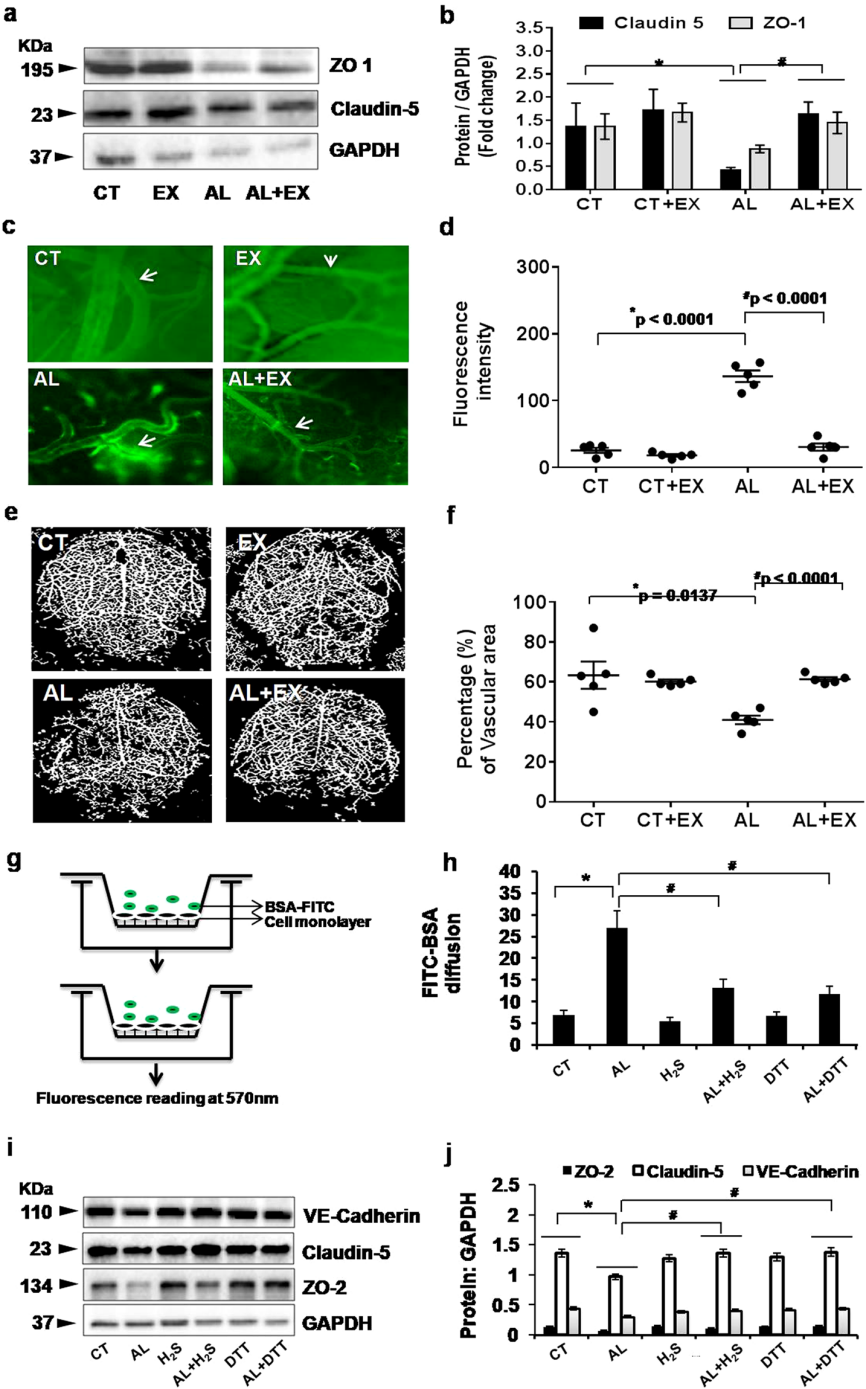
(**a**,**b**) Effect of exercise on alcohol induced vascular permeability and BBB dysfunction. Representative western blot analysis showing the levels of tight junction (TJ) proteins (ZO-1 and Claudin-5) in the different mice groups (**a**). Histogram showing the quantitative estimation of ZO-1 and Claudin-5 proteins after normalization with GAPDH (b). (**c,d**) Representative images showing fluorescent protein (FITC-BSA) leakage from pial vessels into brain parenchyma – indicating alteration in microvascular permeability in the different groups of mice (**c**). Scatter dot plot showing quantitative estimation of fluorescent intensity units (FIU) in the different mice groups after FITC-BSA injection (**d**). (**e**,**f**) Representative images of cerebral angiogram with barium sulfate contrast in experimental mice groups (**e**). Scatter dot plot showing the pattern of vascular density in the form of percentage of vascular area in the different mice groups (**f**). (**g**,**h**) Representative images for the *in vitro* model showing microvascular permeability in brain endothelial cells (bEnd.3 cells) by FITC-BSA diffusion assay. Fluorescence intensity of bovine serum albumin conjugated with FITC (BSA-488) in lower chambers of Transwells was measured by fluorimetry and presented as FIU (**g**). Histogram showing quantitative estimation of FIU in different experimental conditions after FITC-BSA treatment in Transwell chambers (**h**). (**i**,**j**) Representative western blot analysis showing the levels of junctional proteins (VE-Cadherin, Claudin-5 and ZO-2) in different experimental conditions of mouse brain endothelial cells (i). Histograms showing the quantitative estimation of ZO-2, Claudin-5 and VE-Cadherin proteins after normalization with GAPDH (j). All the data are represented as mean values ± standard error (SE) in 5 independent experiments. *^,#^p < 0.05 considered significant.*p < 0.05 vs. CT and ^#^p < 0.05 vs. AL group. Uncropped blots for Fig. 6a,i are presented in Supplementary Fig. 6 and 7.

To determine whether exercised mitigated the blood-brain barrier (BBB) permeability in alcoholic mice, we observed the alternation in the tight junction proteins (ZO-1[zonula occludens-1] and Claudin-5) as well as cerebrovascular permeability in the experiential mice brains. Our results showed that alcohol caused a significant decrease in the protein contents of ZO-1, and Claudin-5 (Fig. 6a,b). This decrease was prevented by regular exercise in the AL+EX group. Intracarotid FITC-BSA (fluorescein isothiocyanate conjugate protein bovine) infusion exemplified the increased macromolecular pial venular permeability in the AL group as compared to CT, but this permeability was normal in the AL+EX group (Fig. 6c,d). We evaluated cerebral vascular density through Barium Sulfate Angiography, and we found that there was a significant decrease in cerebral vascular density in the alcoholic group as compared to the control group as other evidence of increased permeability. However, there was a restoration of vascular patency in the AL+EX group (Fig. 6e,f).

### Inhibition of ER stress attenuates the alcohol induced vascular permeability in mouse brain endothelial cells (bEnd.3 cells)

To investigate the effects of ER stress on vascular permeability, we performed FITC-BSA based vascular permeability assay in *in vitro* condition (Fig. 6g). The bEnd.3 cells were plated in Transwell filters, grown for 2 days and were stimulated with alcohol. As cellular antioxidant H_2_S was elevated during exercise; we treated the cells with H_2_S *in vitro*, to study the alcohol mediated decline in vascular permeability. The results showed that the fluorescence intensity of FITC-BSA, leaked into the lower chambers of alcohol treated bEnd.3 cells in Trans-wells, which was significantly greater, compared to control (Fig. 6h). The treatment with H_2_S (anti-oxidant) ameliorated alcohol induced vascular leakage, as evident from our cell monolayer permeability experiment (Fig. 6h). The increase in vascular permeability in the alcohol treated cells was substantially reduced by administration of ER stress inhibitor (dithiothreitol: DTT) (Fig. 6h). Furthermore, to assess the effects of DTT on tight junction proteins, we measured the levels of ZO-2, Claudin-5 and VE-Cadherin expression in each experimental bEnd.3 cells groups. Western blot analysis showed that the levels of ZO-2 (zonula occludens-2), Claudin-5 and VE-Cadherin (vascular endothelial cadherin) in cytoplasmic protein extracts from bEnd.3 cells were significantly decreased after 24 hours of alcohol treatment as compared with control (Fig. 6i,j). Treatment with either H_2_S or DTT in alcohol treated cells recovered the loss of these tight junction proteins and the expression of all three proteins returned to the control levels (Fig. 6J).

### Therapeutic potential of exercise on alcohol induced neuronal damage recovery

**Figure 7:**
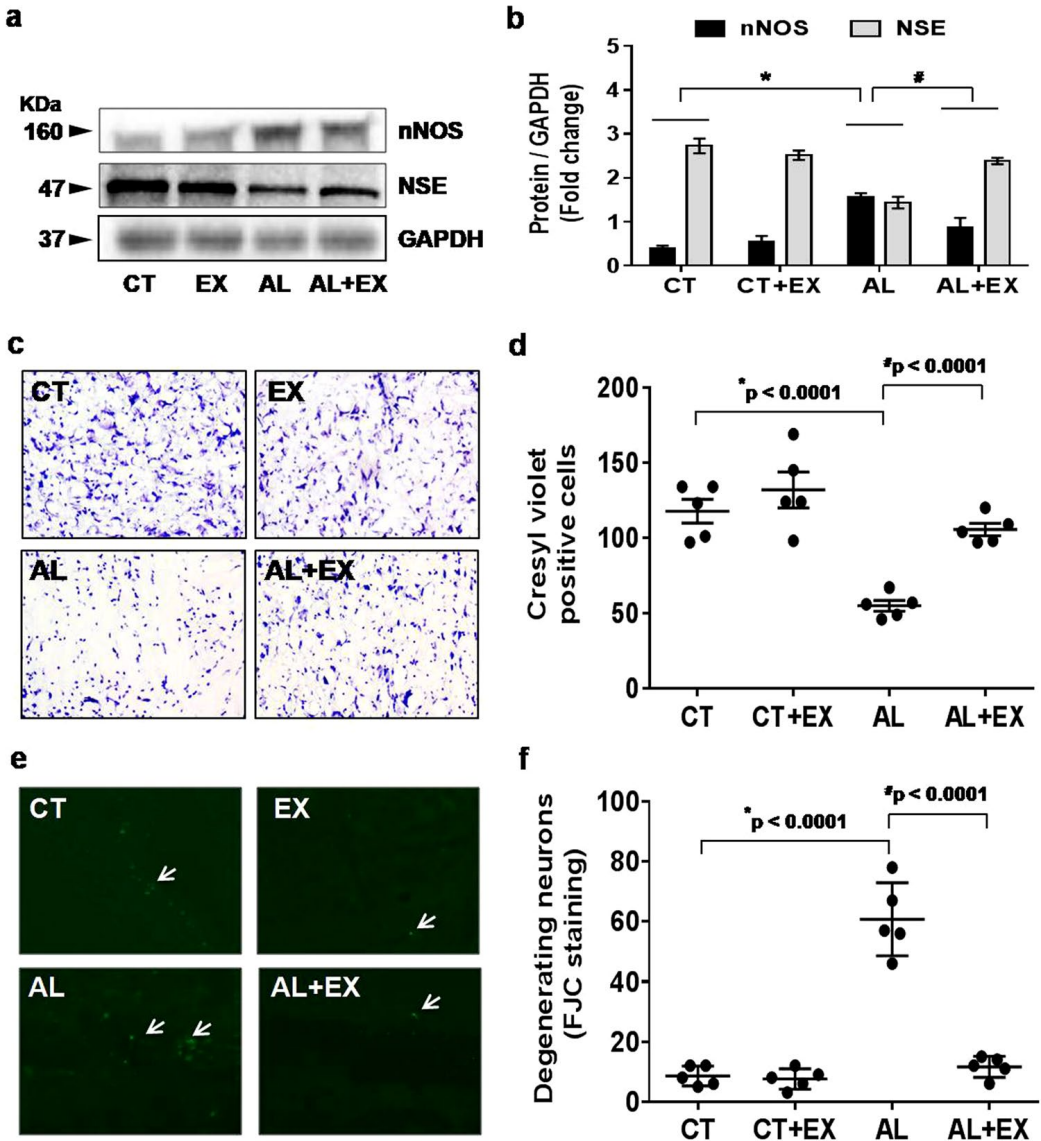
Effect of exercise on alcohol induced neuronal damage. (**a,b**) Representative western blot analysis showing the levels of neuronal proteins (NeuN and NSC) in different mice groups (**a**). Histogram showing the quantitative estimation of nNOS and NSE proteins after normalization with GAPDH (b). (**c,d**) Representative images showing coronal slices of mice brains stained with cresyl violet (40× magnification) (**c**). Scatter dot plot showing the number of cresyl violet positive cells in different groups of mice (**d**). (**e,f**) Representative images showing Fluoro-Jade C (FJC) staining in brain sections of the different groups of mice (10× magnification). A marked decrease of FJC-stained degenerating neurons (arrows) were observed in CT, EX and AL+EX groups, indicating a lesser degree of neuronal cell death. Brain sections of AL treated mice showing a greater number of FJC-positive neurons (arrows), reflecting increased neuronal cell death (**e**). Scatter dot plot showing the numbers of degenerating neurons in different experimental mice groups (**f**). All the data are represented as mean values ± standard error (SE) in 5 independent experiments. *^,#^p < 0.05 considered significant. *p < 0.05 vs. CT and ^#^p < 0.05 vs. AL group. Uncropped blots for a are presented in Supplementary Fig. 8.

To determine the impact of alcohol on the expression of neuronal markers (nNOS [neuronal nitric oxide synthase] and NSE [neuron-specific enolase]), we performed western blot analysis of these proteins in brain tissues of the different experimental mice groups. The results revealed an increased expression of nNOS, reduced expression of NSE in the alcohol treated group compared to the control group, and regular exercise alleviated these effects (Fig. 7a,b).Cresyl violet staining demonstrated a significant loss of cellular constituency as indicated by a decrease in cell size and cell number in the alcohol treated group compared with the control group. However, regular exercise mitigated the alcohol-mediated loss of cellular constituency (Fig. 7c,d). We also performed Flouro-Jade C (FJC) staining to access the neuronal damage and the highest degenerating neurons were observed in the alcohol treated group compared to the control group (Fig. 7e,f). Regular exercise displayed a recovering effect of the neuronal damage. All the above tests confirmed the therapeutic potential of exercise in the recovery of alcohol induced neuronal damage.

## Discussion

Lifestyle patterns such as heavy alcohol consumption and no physical activity are associated with an increased risk of cognitive impairment. The aim of the current study was to characterize the effect of exercise on alcohol-induced neurodegeneration. We demonstrated that ER stress is implicated in the pathogenesis of AL induced BBB dysfunction and cognitive impairment. Exercise-mediated H_2_S restoration; providing novel therapeutics approach for the alcohol-mediated cerebrovascular dysfunction. Accumulating evidence suggested that ER stress is associated with the pathogenesis of several popular neurological diseases such as Huntington’s disease, stroke, Alzheimer’s disease and Parkinson’s disease^52–56^. An involvement of ER stress in alcohol-induced neuron toxicity has been hypothesized. Consistent with these observations, our results showed that cognitive impairment (both short term and long term memory) was significantly decreased in alcohol-treated mice. Moreover, behavioral pattern (spontaneous alternation) of mice was significantly altered in AL group as compared to CT (Fig. 1). But there was no significant difference in the pain sensation between experimental groups vs. controls. Furthermore, exercise markedly reduced the alcohol-induced cognitive dysfunction in AL+EX group compared to CT. These findings suggest that exercise is one of the crucial factors that attenuate alcohol-mediated pathological conditions or cognitive impairments. Other studies reported that maternal ethanol exposure in mice resulted in persistent alternations or decrease in cerebral blood flow^57^. Moreover, decreased cerebral blood flow is associated with cognitive impairment and Alzheimer’s disease as well as other types of dementia^58,59^. Previous reports also suggested the relationship between cerebral blood flow and brain function by showing that an increase in cerebral blood flow is beneficial for cognitive function^60,61^. Concurrent with the above study; we observed a significant decrease in cerebral blood flow and impaired memory function in AL mice brain. Regular practice of exercise significantly improved cerebral blood flow and memory functions in our study (Fig. 2).

HHcy is one of the risk factors for cerebrovascular complications^17,21^. Other investigations have shown that alcohol consumption is closely associated with elevated Hcy levels^62^. Elevated plasma Hcy is associated with an increased risk of vascular disease and vascular dementia (Lehmann *et al*.)^14^. Studies on human subjects reported an increase in blood Hcy levels in patients with Alzheimer’s disease and mild cognitive impairment^15–17^. We also found that exercise significantly reduced the total plasma Hcy in the AL group as compared to CT group (Fig. 3). Increased plasma concentration of Hcy is considered as a risk factor for vascular disease, brain atrophy and a decline in memory scores^20,63^. Several follow up studies demonstrated a positive association between the level of Hcy and cognitive functions^17^. We found that the alcohol treatment induced the alteration in the expression of enzymes involved in Hcy-metabolism such as CBS, CSE, MTHFR, and SAHH (Fig. 3). In particular, Hcy levels are increased in the body when metabolism of cysteine or methionine is impaired. So our results clearly showed that alcohol interfered with the methionine metabolism directly or indirectly and altered the methionine metabolizing enzymes that led to HHcy. However, the regular practice of exercise ameliorated these changes. Overall, our data suggest that an increased level may play a significant role in the altered expression of methionine metabolizing enzymes and the protective role of exercise in this matter.

Exercise-mediated increased expression of CBS, CSE enzymes were observed in the EX group that are involved in the generation of endogenous hydrogen sulfide (H_2_S). Co-current this observation we found increased levels of H2S in AL+EX group as compared to AL group (Fig. 4). H_2_S can easily penetrate the plasma membrane thus inducing a wide spectrum of signaling cascades in the target cells. Endogenous H_2_S acts as a potent regulator of various biological processes especially related to vasomotor function, anti-oxidant and anti-inflammatory responses. Redox stress has been proposed to be a major contributor to alcohol-induced neurotoxicity^64^. Other study showed that six weeks of ethanol consumption caused the elevation of oxidative stress in rats^65^. The results of the current study showed that alcohol consumption significantly increased the NOX-4 protein, MDA and lipid peroxidation (oxidant) products. In contrast decreased the catalase protein as well as glutathione peroxidase (GPx) antioxidant activity. The imbalance in oxidant vs antioxidant levels observed in this study is consistent with our recent similar findings in hippocampus and aorta tissue of ethanol-exposed rats and rules out its involvement in raising the ROS level^66^. Our results vividly demonstrated that exercise boosts the antioxidant system (both H_2_S level and GPx activity) that helped alleviate the alcohol-mediated oxidative stress.

Alcohol-induced endoplasmic reticulum (ER) stress, which can be caused by acetaldehyde, adducts production, oxidative stress, and disturbed intracellular calcium homeostasis, also participates in alcohol-induced neurotoxicity^64^. ER stress has been suggested to be the cause of a wide range of cellular dysfunction involving accumulation of unfolded or misfolded proteins in the ER under alcohol exposure in both *in-vitro* and *in-vivo*^53–55,67^. The accumulation of unfolded or misfolded proteins in the ER lumen induces unfolded protein response (UPR) that triggers ER stress, which is mediated by transmembrane ER signaling proteins. Consistent with these observations, we observed ER stress-mediated increased expression of GRP 78 and ATF6 protein and excess release of Ca^+2^ in the alcohol-treated mice and exercise-mediated normalized expression in the AL+EX mice group(Fig. 4). The present study also indicated that, the ER stress response protein ATF6 become upregulated and upon entering into the nucleus binds to the CpG islands of Herp promoter leading to Herp upregulation (Fig. 5). Moreover, we have analyzed the effect of alcohol on DNMT1 and DNMT3, as studies suggested potential role of these molecules on neural development under alcoholism and stress^68^. Other study showed that alcohol exposure upregulate DNMT3a protein levels in neural precursor and primary mouse embryonic cell lines and this upregulation may be linked with generation of ROS^69^. Contrary to the previous report on cell line study; our animal experiments revealed that, DNMT1 and DNMT3a protein levels were significantly decreased in brain tissue of mice under alcoholism and exercise restored their levels back to normal (Fig. 5).

Several tight junction proteins (TJPs), such as Claudins and ZOs help to maintain the structure/integrity of blood brain barrier. Previous studies have indicated that alterations in ZO-1 and Claudin-1 are associated with increased intestinal permeability and a higher susceptibility to alcoholic liver disease^70^. Similarly, our study shows that alcohol exposure resulted in down-regulation and/or redistribution of ZO-1 and Claudin-5 proteins suggesting that these TJPs are critical in alcohol-induced blood brain barrier dysfunction (Fig. 6). Next, we confirmed blood brain barrier integrity by correlating the expression of TJPs directly with brain vessel permeability in mice. The intravital fluorescence microscopy analysis revealed increased brain pial venular permeability in AL group as compared to CT (Fig. 6). In addition, we have also measured diminished vessel density in alcoholic group brain suggesting that vascular density is decreased in alcoholic group vs control. To strengthen our hypothesis, we administered Dithiothreitol (DTT), a potent ER stress inhibitorin- *vitro* to conclusively show the role of ER stress in regulation of the BBB permeability We observed that DTT reduced vascular permeability in brain endothelial cells *in- vitro* (Fig. 6). Collectively, our data suggested that inhibition of ER stress could potentially reduce cerebrovascular permeability and tight junction proteins alternations. Additionally, therapeutic potential of regular practice of exercise in the recovery of alcohol induced neuronal damage has also been shown in our current study (Fig. 7).

**Figure 8:**
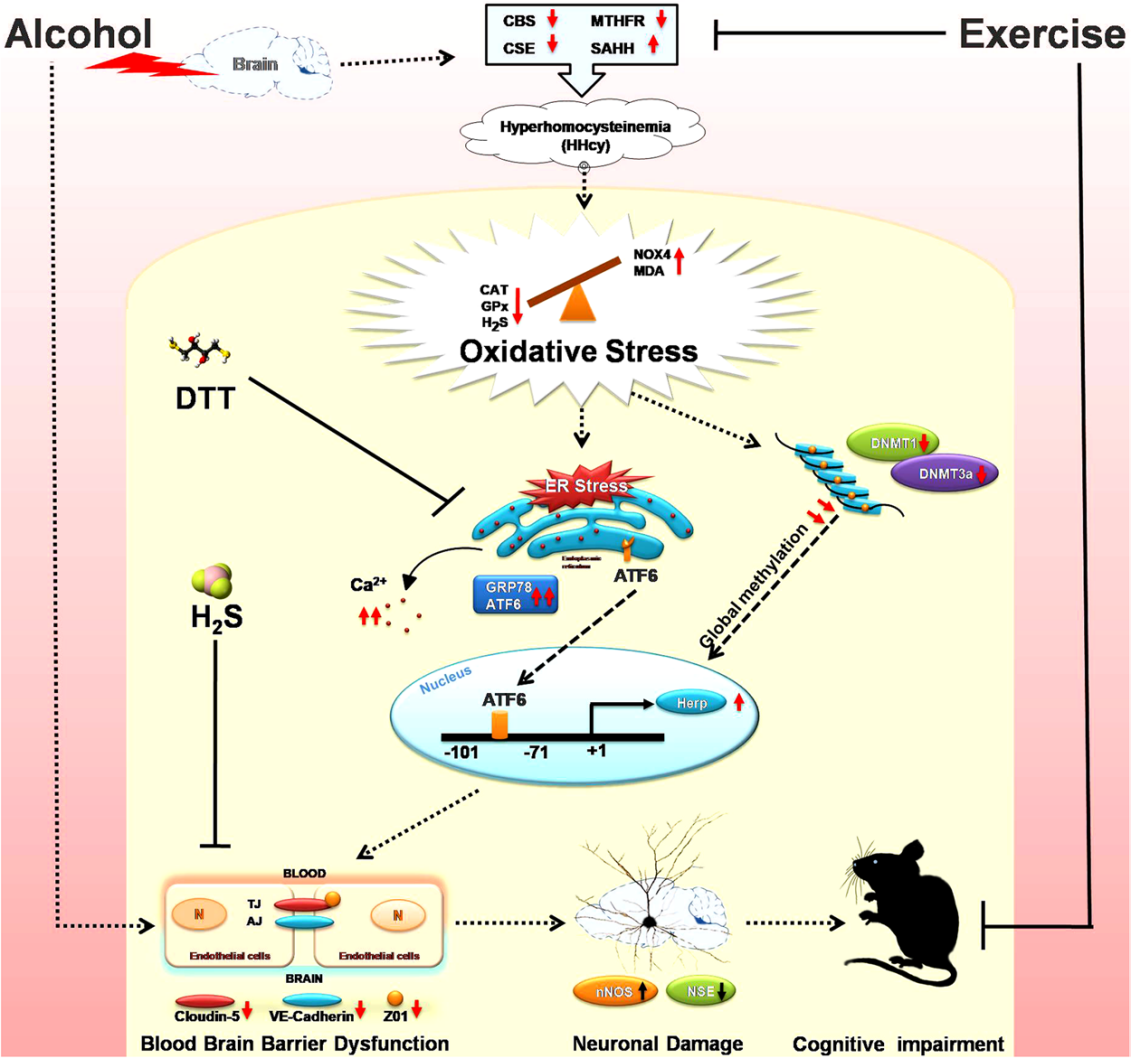
Diagrammatic representation of the overall study finding and its relation to cognitive impairments.

Summary of theresearch finding is presented in Fig. 8. Our study clearly indicates that alcohol can exert its effect on the brain and subsequent alteration in homocysteine metabolizing enzymes leading to hyperhomocysteinemia (HHcy). HHcy mediated disturbed redox homeostasis subsequently elevated ER stress and global methylation by altering the DNMT activities. ER stress response protein ATF6 become upregulated and upon entering the nucleus binds to CpG islands of Herp promoter leading to Herp upregulation. This alteration may induce blood-brain barrier dysfunction through down-regulation of TJPs presumably leading to neuronal damage and cognitive impairments (Fig. 8). Thus, our results showed that pathogenesis of alcohol-induced cerebrovascular dysfunction and memory impairment are associated with ER stress. Exercise-mediated enhanced biosynthesis of the H_2_S and GPx thus activates the cellular antioxidant system and subsequently alleviate these effects. Therefore, exercise intervention could serve as a powerful preventive and therapeutic approach that can havea tremendous improvements physiological function. Moreover, regular practice of exercise may also be responsible for the interruption of the ER stress and again is anticipated to have therapeutic potential for the better management of alcohol-related diseases or disorders.

## Methods

### Antibodies

Antibodies like CBS, CSE, MTHFR, Hcy, CAT, NOX4, GRP78, ATF6, DNMT1, DNMT3a and Herp were all obtained from Abcam (Cambridge, MA USA). Other antibodies viz. VE-Cadherin, nNOS, NSE, GAPDH were from Boster Biological Technology (Pleasanton, CA, USA). Antibodies like *Zonula occludens-1 (ZO-1)*, SAHH, Claudin-5 were purchased from Santa Cruz Biotechnology (Santa Cruz, CA, USA).

### Animal model

Wild-type male mice (strain: C57BL/6 J; age: 8–10 weeks; weight: 29–32 gms.) were obtained from Jackson Laboratory (Bar Harbor, ME) and kept in a suitable environmental condition (12:12-h light-dark cycle, 22–24 °C) at the animal care facility of our University of Louisville School of Medicine. All mice were given ad libitum access to standard chow and water. All animal procedures were reviewed and approved by the Institutional Animal Care and Use Committee (IACUC) at the University of Louisville School of Medicine. Moreover, methods and general guidelines for animal use were followed according to the Animal Care and Use Program Guidelines of the National Institutes of Health.

### Exercise protocol

Selected groups of experimental mice were exercised using a small animal treadmill (#1050 RM-E57) from Columbus Instruments International, with zero inclination^71^. The initial adaptation was introduced at a slow speed of 7 m/min for 5 days. Later, the mice were monitored to cover a daily distance of 330 m with a speed of 10 m/min for the first two weeks and then a speed of 11 m/min for the remaining period of 3 weeks, 5 days/week for a total of 12 weeks. After every 110 m for the entire exercise period and at each incremental speed, the mice were given rest for 10 minutes. Active monitoring was conducted to ensure mice movement and safety.

### Experimental design and drug administration

The mice (n = 5 for each study) were grouped as following:

(i) CT (control group): Wild-type male mice without alcohol administration and exercise treatment.
(ii) AL (alcohol treated group): Wild-type male mice with the alcohol (ethanol) administration (1.5 g.kg^−1^ day^−1^ for 5 days/week).
(iii) CT+EX (control with exercise group): Wild-type male mice without any alcohol administration but daily exercise continued for 12 weeks. CT+EX groups were injected with normal saline at similar dose like alcohol.
(iv) AL+EX (alcohol treated with exercise group): Wild-type male mice with alcohol administration and daily exercise continued for 12 weeks.

### Assessment of behavior and memory: NORT, PAT and Y-maze tests

To assess memory related behaviors, the novel object recognition test (NORT), Passive Avoidance Task (PAT) and Y-maze (spontaneous alternation and two-trial recognition) tests were performed according to our previously published literature and other investigators^72–76^. In our study, the NORT was used to test short-term memory, the PAT evaluated long-term memory and the Y-maze test assessed spatial working short-term memory in all experimental mice.

### Cortical blood flow and blood pressure measurement

Under intraperitoneal tribromoethanol (*TBE @ 5 mg/kg bw*) anesthesia, each animal was placed in the prone position, and the brain blood flow was measured using Speckle Contrast Imager (Moor FLPI, Wilmington, DE) at room temperature. The camera (580 9 752 resolution) was positioned 15 cm from the dorsal surface of the brain. Settings for lower solution/high-speed images included a display rate of 25 Hz, the time constant of 1.0 sec, and a camera exposure time of 20 m/sec. The contrast images were processed to produce a color-coded live flux image and a flux unit’s trace was also recorded for 2 min in all of the animals. Data were expressed as flux unit. On the other hand, blood pressure (BP) in the conscious animal was measured by a non-invasive tail-cuff method (CODA 2, Kent Scientific Corp., Torrington, CT) after proper acclimatization in a steady state^77^.

### Estimation of biochemical parameters: tHcy, MDA, GPx, H_2_S, CBS & DNMT activity, Ca^2+^

At the end of each experiment, blood was withdrawn by venipuncture of the vena cava using a 23-gauge needle and polypropylene syringe containing sodium citrate. Blood was transferred to Eppendorf tubes, centrifuged at 1000 × g for 15 min to obtain supernatant plasma. Brain tissues from different experimental mice groups were also collected and homogenized in 0.1 M phosphate buffer (pH 7.4). The level of total homocysteine (tHcy) in plasma samples was measured by a mouse homocysteine Kit manufactured by Crystal Chem, Chicago, IL, USA, Catalog No.80440 according to their protocol. Lipid peroxide product malondialdehyde (MDA) in the brain was estimated according to the previously described protocol and was expressed as µmol/mg of protein^78^. The activity of glutathione peroxidase (GPx) was evaluated by colorimetric assay using the guidelines for a commercial kit from Abcam (GPx Activity Kit; ab102530). The activity of glutathione peroxidase was expressed as U/mg of protein according to the manufacturer’s instructions. Production of hydrogen sulfide (H_2_S) from brain tissue homogenate was measured following the protocol by Qu *et al*., 2006 and the data were expressed as nmol/mg of protein^79^. Details of the experimental protocols can be available in our previously published article^72^. Cystathionine β-synthase (CBS) activity assay was performed in the brain homogenate according to the prior published literature^80^. CBS activity data were expressed as nmol/min/mg protein. *DNA Methyltransferase* (DNMT) activity was ascertained according to our previously published protocol^81^, in which nuclear protein fractions were isolated from the mouse brain using EpiQuik™ nuclear extraction kit (Epigentek, Farmingdale, NY, USA). DNMT activity was assessed through an EPiQuik™ DNMT activity/inhibitor assay ultra kit (Epigentek, Farmingdale, NY, USA) according to the manufacturer’s instruction. DNMT activity data were expressed as OD/h/mg of protein. Measurement of Ca^2+^was done by the use of commercially available Calcium Assay Kit (MAK022-1KT) purchased from Sigma. The levels of Ca^2+^ were expressed as mM/L according to manufacturer’s protocol.

### Collection and preparation of brain protein

Preparation of brain tissue samples was followed by our previously published standard protocol^82^. In brief, brain samples were weighed and homogenized in 1× RIPA buffer (Tris–HCl 50 mM, pH 7.4; NP-40, 1%; 0.25% Na-deoxycholate, 150 mM NaCl; 1 mM EDTA; 1 mM PMSF; 1 μg/ml each of aprotinin, leupeptin, pepstatin; 1 mM Na3VO4; 1 mM NaF) containing 1 mM PMSF and 1 μg of complete protease inhibitor (Sigma). After two cycles of centrifugation, supernatant protein levels for all samples were quantified by the Bradford method (Bio-Rad, CA) and stored at −80 °C for further use.

### Western blot analysis

Western blot analysis for the homocysteine metabolized enzymes (CBS, CSE, MTHFR, SAHH and Hcy), markers of oxidative stress (CAT, NOX4), hallmarks of ER stress (GRP78, ATF6), markers of DNA methylation (DNMT1, DNMT3a), homocysteine-induced endoplasmic reticulum protein (Herp) and tight junction proteins (ZO1, Claudin-5) were performed by following the standard procedure previously published^82^. The relative optical density of protein bands was analyzed using gel software Image Lab 3.0. The membranes were stripped and re-probed with GAPDH as a loading control.

### RNA isolation and real-time PCR

Total RNA was isolated from the mouse brain using Invitrogen TRIzol Reagent according to the manufacturer’s instructions. The cDNA was synthesized from total RNA using ImProm-II™ Reverse Transcription system kit (Promega Corporation, Madison, WI, USA). RT-qPCR was performed using the Roche LightCycler 480 Real-Time PCR according to the manufacturer’s instructions. GAPDH was used as the internal control. The primer sequences employed are as follows: The mRNA levels of CBS, CSE, MTHFR, SAHH and GAPDH were determined by RT-PCR using the following primers: (i) CBS: NM 144855 (567 bp)- Forward: 5′-AGG GCT ATC GCT GCA TTA TCG TGA-3′& Reverse: 5′-AGC TTC CAC CAC ATA GCA GTC CTT-3′; (ii) CSE: NM_145953 (304 bp)- Forward: 5′-CAAAGCAACACCTCGCACTC-3′& Reverse: 5′-CAGCAAGACCCGATGCAAAG-3′; (iii) MTHFR: NM_001161798.1 (85 bp)- Forward: 5′-AGGACGGTGCGGTGAGAGTG-3′& Reverse: 5′-TGAAGGAGAAGGTGTCTGCGGGA-3′; (iv) SAHH: NM_016661.3 (83 bp)- Forward: 5′-CACCAGATGTCCCATCGCTT-3′& Reverse: 5′-GGGAAGAGCAGAAATGGCCT-3′; (v) GAPDH: NM_001289726.1 (87 bp)- Forward: 5′-TGCACCACCAACTGCTTTGC-3′& Reverse: 5′-GGCATGGACTGTGGTCATGAG-3′.

### Chip assay

The chromatin Immunoprecipitation (ChIP) assay was performed as per previously published protocol^83^. Briefly, after cross-linking in 4% formaldehyde, the brain tissues were lysed. The supernatant was precleared with protein A beads. Equal amounts of samples were used in the immunoprecipitation. A 5–7% aliquot of the precleared chromatin was taken as input, and the remaining samples were immunoprecipitated with the anti-ATF6 monoclonal antibody (Abcam, Cambridge, MA USA).The precipitated samples were used in a PCR to produce a 179-bp Herp product with the primers: Forward: 5′-CAGACGCGGCGGGTTGCA-3′ and Reverse: 5′-GCTTCGGGCGCCTTTTATAGA-3′ for the endogenous Herp promoter and input DNA used as control ChIP assays. The gel electrophoresis images were acquired on a ChemiDoc™ XRS+System with Image Lab™ Software after ethidium bromide staining.

### Microvascular leakage observation by intravital microscopy

Blood flow in brain microvasculature was measured in anesthetized mice as described earlier^84^. Briefly, pial brain microcirculation was prepared for observation according to the method of Muradashvili *et al*.^85^. A mixture of 100 μl of FITC (300 μg/ml) and 20 μl of BSA-488 (3.3 mg/ml) in PBS was injected through the carotid artery cannulation and allowed to circulate for up to 10 min. *In vivo* imaging with a BX61WI fluorescent microscope (Olympus, Tokyo, Japan) was used to examine the exposed area of the skull. Data were interpreted with the software provided with the instrument and Image-Pro Plus 6.3 software (Media Cybernetics, Bethesda, MD).

### Barium sulfate angiography

Barium sulfate angiography was performed in mice as described in our previously published procedure^82^. Barium sulfate (0.1 g/mL) was dissolved in 50 mM Tris-buffer (pH 5.0) and infused slowly at a constant flow and pressure with a syringe pump through the carotid artery^86,87^. The brain was dissected out and placed in the X-ray chamber Kodak 4000 MM image station and angiograms were captured with the high penetrative phosphorous screen by 31 KVP X-ray exposures for 3 minutes. Brain vessel density was quantified using Vessel Segmentation and Analysis (VesSeg) software tool (http://www.isip.uni-luebeck.de/index.php?id=150) according to the procedure of Pushpakumar *et al*.^88^.

### *In vitro* endothelial cell (bEnd.3) permeability assays

The vascular permeability was measured according to the protocol described in previous literature in detail (Nooteboom *et al*.)^89^. To study endothelial permeability *in vitro*, the bEnd.3 cells were seeded on the Transwell filters containing a tracer solution with FITC-BSA (200 µg/ml) and subjected to respective treatments in different experimental groups (CT, AL, H_2_S, AL+H_2_S, DTT and AL+DTT). Following treatments, the culture medium was replaced by 0·5 ml of the tracer solution at the upper side of the monolayer. After 2 h of incubation, 100 µl of each sample was drawn from the lower compartment and transferred to 96-well microtiter plates. For the detection of FITC-BSA, microtiter plates were read on Max3000 plate reader (Molecular Devices) at 750 nm. Data were expressed as fluorescence intensity unit (FIU) in each group.

### Preparation of brain sections for microscopy

Brains from different mice groups were carefully removed, stored in 4% paraformaldehyde (PFA) overnight, transferred to a 30% sucrose solution for 3–5days then mounted in a protective (OCT) matrix (Polyscience, Inc., Warrington, PA, USA). The brains were then cryo-sectioned using a Leica CM 1850 cryostat (Bannockburn, IL, USA) into 20–25 μm thick slices^90^ and stored at −80 °C until further analysis for cresyl violet and FJC staining.

### Cresyl violet staining

Cresyl violet staining was done as per standard protocol^81^. In brief, brain sections (15 μm) were stained with cresyl violet dye for 5 min, air-dried for 1 h and dipped in *n*-butanol for 2 min, acetone and xylene for 10 min each, and mounted in DPX. The images were acquired using a fluorescent microscope at ×20 magnification. The cellular morphology was checked in the brain sections and the numbers of cresyl violet positive cells were counted.

### Fluoro-Jade C staining

Fluoro-Jade C (FJC) labeling in the brain sections was performed using a standard protocol^82^. Briefly, 25 μM cryo-sectioned brain tissues were deparaffinized by two 5-min washes in xylene, rehydrated through a graduated alcohol series (100, 90, 70, 50, 30%) each for 5 min and finally washed for 2 min in distilled water. The sections were then transferred to 0.06% potassium permanganate solution for 10 min and rinsed in distilled water for 2 min. Thereafter, the sections were incubated for 20 min in a 0.0001% solution of FJC (Sigma Aldrich). FJC was made immediately before use by diluting a stock solution of 0.01% FJC by 100-fold in 0.1% acetic acid. The sections were eventually dried at 37 °C and mounted with DPX. The fluorescent signal was visualized using a *fluorescence microscope* with an excitation wavelength of 488 nm.

### Statistical analysis

Data analyses and graphical presentations were performed with GraphPad InStat 3 and GraphPad Prism, version 6.07 (GraphPad Software, Inc., La Jolla, CA). Data are represented as mean values ± standard error (SE) in five independent experiments in all cases. The experimental groups were compared by one-way analysis of variance (ANOVA) assuming that the values were sampled from Gaussian distributions. For a set of data, if ANOVA indicated a significant difference (p < 0.05); Tukey-Kramer multiple comparison tests were used to compare group means. Post-test was only performed if p < 0.05. If the value of Tukey-Kramer ‘q’ is less than 4.046, then the p-value is less than 0.05 and considered statistically significant.

### Data Availability

All data generated or analyzed during this study are included in this published article.

## Electronic supplementary material

Supplementary information

## References

[1] Di Castelnuovo A (2006). Alcohol dosing and total mortality in men and women: an updated meta-analysis of 34 prospective studies. Arch Intern Med.

[2] Seitz HK, Stickel F (2007). Molecular mechanisms of alcohol-mediated carcinogenesis. Nat Rev Cancer.

[3] Boffetta P, Hashibe M (2006). Alcohol and cancer. Lancet Oncol.

[4] Nelson DE (2013). Alcohol-attributable cancer deaths and years of potential life lost in the United States. Am J Public Health.

[5] Ronksley PE (2011). Association of alcohol consumption withselected cardiovascular disease outcomes: a systematic review and meta-analysis. BMJ.

[6] Thompson PL (2013). J-curve revisited: cardiovascular benefits of moderate alcohol usecannot be dismissed. Med J Aust..

[7] NIAAA National Institute on Alcohol Abuse and Alcoholism No. 34 PH 370 October 1996. Alcohol alert. Availabe at NIAAA's World Wide Web site at http://www.niaaa.nih.gov.

[8] Shayakhmetova GM, Bondarenko LB, Matvienko AV, Kovalenko VM (2014). Chronic alcoholism-mediated metabolic disorders in albino rat testes. InterdiscipToxicol..

[9] Skovierova H (2016). The Molecular and Cellular Effect of Homocysteine Metabolism Imbalance on Human Health. Int J Mol Sci..

[10] Ji C, Kaplowitz N (2004). Hyperhomocysteinemia, endoplasmic reticulum stress, and alcoholic liver injury. World J Gastroenterol..

[11] Gibson JV (2008). Alcohol increases homocysteine and reduces B vitamin concentration in healthy male volunteers—a randomized, crossover intervention study. QJM..

[12] Fowler AK (2012). Alcohol-induced one-carbon metabolism impairment promotes dysfunction of DNA base excision repair in adult brain. J Biol Chem..

[13] Bleich S, Degner D, Javaheripour K, Kurth C, Kornhuber J (2000). Homocysteine and alcoholism. J Neural Transm Suppl..

[14] Lehmann M, Gottfries CG, Regland B (1999). Identification of cognitive impairment in the elderly: homocysteine is an early marker. DementGeriatrCogn Disord..

[15] McCaddon A (1998). Total serum homocysteine in senile dementia of Alzheimer type. Int J Geriatr Psychiatry..

[16] Clarke R (1998). Folate, vitamin B12, and serum total homocysteine levels in confirmed Alzheimer disease. Arch Neurol..

[17] Ravaglia. G (2003). Homocysteine and cognitive function in healthy elderly community dwellers in Italy. Am J ClinNutr..

[18] McCaddon A (2001). Homocysteine and cognitive decline in healthy elderly. Dement GeriatrCognDisord..

[19] Tucker KL, Qiao N, Scott T, Rosenberg I, Spiro A (2005). High homocysteine and low B vitamins predict cognitive decline in aging men: the Veterans Affairs Normative Aging Study. Am J ClinNutr..

[20] Nurk E (2005). Plasma total homocysteine and memory in the elderly: the Hordaland homocysteine study. Ann Neurol..

[21] Seshadri S (2002). Plasma homocysteine as a risk factor for dementia and Alzheimer's disease. N Engl J Med..

[22] Bravo (2013). Endoplasmic Reticulum and the Unfolded Protein Response: Dynamics and Metabolic Integration. Int Rev Cell Mol Biol..

[23] Salaroglio IC (2017). PERK induces resistance to cell death elicited by endoplasmic reticulum stress and chemotherapy. Mol Cancer..

[24] Hoozemans JJ (2005). The unfolded protein response is activated in Alzheimer’s disease. ActaNeuropathol..

[25] Hoozemans JJ (2009). The unfolded protein response is activated in pretangle neurons in Alzheimer's disease hippocampus. J Pathol..

[26] Stutzbach LD (2013). The unfolded protein response is activated in disease-affected brain regions in progressive supranuclear palsy and alzheimer’s disease. ActaNeuropathol Commun..

[27] Zeeshan HM (2016). Endoplasmic Reticulum Stress and Associated ROS. Int J Mol Sci..

[28] Zou CG, Banerjee R (2005). Homocysteine and redox signaling. Antioxid Redox Signal..

[29] Ho PI (2001). Homocysteine potentiates beta-amyloid neurotoxicity: role of oxidative stress. J Neurochem..

[30] Reis EA (2002). Pretreatment with vitamins E and C prevent the impairment of memory caused by homocysteine administration in rats. Metab. Brain Dis..

[31] Hosoki R, Matsuki N, Kimura H (1997). The possible role of hydrogen sulfide as an endogenous smooth muscle relaxant in synergy with nitric oxide. BiochemBiophys Res Commun..

[32] Kohn C, Dubrovska G, Huang Y, Gollasch M (2012). Hydrogen sulfide: potent regulator of vascular tone and stimulator of angiogenesis. Int J Biomed Sci..

[33] Zhao H, Chen MH, Shen ZM, Kahn PC, Lipke PN (2001). Environmentally induced reversible conformational switching in the yeast cell adhesion protein alpha-agglutinin. Protein Sci..

[34] Asimakopoulou A (2013). Selectivity of commonly used pharmacological inhibitors for cystathionine β synthase (CBS) and cystathionine γ lyase (CSE). Br J Pharmacol..

[35] Sen U (2012). Increased endogenous H2S generation by CBS, CSE, and 3MST gene therapy improves *ex vivo* renovascular relaxation in hyperhomocysteinemia. Am J Physiol Cell Physiol..

[36] Kimura H (2014). Production and Physiological Effects of Hydrogen Sulfide. Antioxid Redox Signal..

[37] Calvert, J. W. *et al*. Genetic and pharmacologic hydrogen sulfide therapy attenuates ischemia-induced heart failure in mice. *Circulation*. 12211–19 (2010). 10.1161/CIRCULATIONAHA.109.920991 PMC2955293 20566952

[38] Yang G, Yang W, Wu L, Wang R (2007). H_2_S, endoplasmic reticulum stress and apoptosis of insulin-secreting beta cells. J Biol Chem..

[39] Wang S, Kaufman RJ (2012). The impact of the unfolded protein response on human disease. J Cell Biol..

[40] Salmina AB (2015). H2S- and NO-Signaling Pathways in Alzheimer's Amyloid Vasculopathy: Synergism or Antagonism?. Front Physiol..

[41] Bir (2012). Hydrogen Sulfide Stimulates Ischemic Vascular Remodeling Through Nitric Oxide Synthase and Nitrite Reduction Activity Regulating Hypoxia‐Inducible Factor‐1α and Vascular Endothelial Growth Factor–Dependent Angiogenesis. J Am Heart Assoc..

[42] Kimura Y, Dargusch R, Schubert D, Kimura H (2006). Hydrogen sulfide protects HT22 neuronal cells from oxidative stress. Antioxid Redox Signal..

[43] Kimura Y, Kimura H (2004). Hydrogen sulfide protects neurons from oxidative stress. FASEB J..

[44] Shiroma EJ, Lee IM (2010). Physical activity and cardiovascular health: lessons learned from epidemiological studies across age, gender, and race/ethnicity. Circulation.

[45] Sofi F, Capalbo A, Cesari F, Abbate R, Gensini GF (2008). Physical activity during leisure time and primaryprevention of coronary heart disease: an updated meta-analysis of cohort studies. Eur J CardiovascPrevRehabil..

[46] Sadarangani KP, Hamer M, Mindell JS, Coombs NA, Stamatakis E (2014). Physical activity and risk of all-causeand cardiovascular disease mortality in diabetic adults from Great Britain: pooledanalysis of 10 population-based cohorts. Diabetes care.

[47] Hamer M (2012). Physical activity and cardiovascular mortality risk:possible protective mechanisms?. Med Sci Sports Exerc..

[48] Perreault K (2017). Does physical activity moderate the association between alcohol drinking and all-cause, cancer and cardiovascular diseases mortality? A pooled analysis of eight British population cohorts. Br J Sports Med..

[49] Konig D (2003). Influence of Training Volume and Acute Physical Exercise on the Homocysteine Levels in Endurance-Trained Men: Interactions with Plasma Folate and Vitamin B12. Ann Nutr Metab..

[50] Winchester. L, Veeranki S, Givvimani S, Tyagi SC (2014). Exercise mitigates the adverse effects of hyperhomocysteinemia on macrophages, MMP-9, skeletal muscle, and white adipocytes. Can J Physiol Pharmacol..

[51] Liang (2006). Luman/CREB3 induces transcription of the endoplasmic reticulum (ER) stress response protein Herp through an ER stress response element. Mol Cell Biol..

[52] Tajiri S (2004). Ischemia-induced neuronal cell death is mediated by the endoplasmic reticulum stress pathway involving CHOP. Cell Death Differ..

[53] Katayama T (2004). Induction of neuronal death by ER stress in Alzheimer's disease. J ChemNeuroanat..

[54] Silva RM (2005). CHOP/GADD153 is a mediator of apoptotic death in substantia nigra dopamine neurons in an *in vivo* neurotoxin model of parkinsonism. J Neurochem..

[55] Scheper W, Hoozemans JJ (2009). Endoplasmic reticulum protein quality control in neurodegenerative disease: the good, the bad and the therapy. Curr Med Chem.

[56] Stampfer MJ, Kang JH, Chen J, Cherry R, Grodstein F (2005). Effects of Moderate Alcohol Consumption on Cognitive Function in Women. N Engl J Med.

[57] Bake S (2012). Ethanol exposure during pregnancy persistently attenuates cranially directed blood flow in the developing fetus: evidence from ultrasound imaging in a murine second trimester equivalent model. Alcohol Clin Exp Res..

[58] O'Brien JT, Eagger S, Syed GM, Sahakian BJ, Levy R (1992). A study of regional cerebral blood flow and cognitive performance in Alzheimer's disease. J Neurol Neurosurg Psychiatry..

[59] Prohovnik I (1988). Cerebral perfusion as a diagnostic marker of early Alzheimer's disease. Neurology.

[60] Nash DT, Fillit H (2006). Cardiovascular disease risk factors and cognitive impairment. Am J Cardiol..

[61] Tota S (2009). Candesartan improves memory decline in mice: involvement of AT1 receptors in memory deficit induced by intracerebral streptozotocin. Behav Brain Res..

[62] Cheng J, Kaplowitz N (2004). Hyperhomocysteinemia, endoplasmic reticulum stress, and alcoholic liver injury. World J Gastroenterol.

[63] Den Heijer T (2003). Homocysteine and brain atrophy on MRI of non-demented elderly. Brain.

[64] Li J, Cheng J (2017). Apolipoprotein E4 exacerbates ethanol-induced neurotoxicity through augmentation of oxidativestress and apoptosis in N2a-APP cells. Neurosci Lett..

[65] Shirpoor A (2016). Protective effect of vitamin E against ethanol-induced small intestine damage in rats. Biomed Pharmacother..

[66] Kamat PK, Kyles P, Kalani A, Tyagi N (2016). HydrogenSulfideAmelioratesHomocysteine-InducedAlzheimer'sDisease-LikePathology, Blood-BrainBarrierDisruption, and SynapticDisorder. Mol Neurobiol..

[67] Ke Z (2011). Ethanol induces endoplasmic reticulum stress in the developing brain. Alcohol Clin and Exp Res..

[68] Gräff J, Kim D, Dobbin MM, Tsai LH (2011). Epigenetic regulation of gene expression in physiological and pathological brain processes. Physiol Rev..

[69] Miozzo F (2018). Alcohol exposure promotes DNA methyltransferase DNMT3A upregulation through reactive oxygen species-dependent mechanisms. Cell Stress Chaperones..

[70] Wang Y (2014). Effects of alcohol on intestinalepithelialbarrierpermeability and expression of tight junction-associatedproteins. Mol Med Rep..

[71] Veeranki S (2016). Moderateintensityexercisepreventsdiabeticcardiomyopathyassociatedcontractiledysfunction through restoration of mitochondrialfunction and connexin43levels in db/dbmice. J Mol Cell Cardiol..

[72] Kamat PK, Tota S, Saxena G, Shukla R, Nath C (2010). Okadaic acid (ICV) induced memory impairment in rats: a suitable experimental model to test anti-dementia activity. Brain Res..

[73] Lyon DR, Gunzelmann GM (2011). Functional equivalence and spatial path memory. Q J Exp Psychol..

[74] Pinel JPJ (1968). Evaluation of the one-trial passive avoidance task as a tool for studying ECS-produced amnesia. Psychonom Sci..

[75] Kutiyanawalla A, Promsote W, Terry A, Pillai A (2012). Cysteamine treatment ameliorates alterations in GAD67 expression and spatial memory in heterozygous reeler mice. Int J Neuropsychopharmacol..

[76] Jadavji NM, Deng L, Malysheva O, Caudill MA, Rozen R (2015). MTHFR deficiency or reduced intake of folate or choline in pregnant mice results in impaired short-term memory and increased apoptosis in the hippocampus of wild-type offspring. Neuroscience.

[77] Nithya N (2014). Epigenetic regulation of aortic remodeling in hyperhomocysteinemia. FASEB J..

[78] Okhawa H, Oshishi N, Yag K (1979). Assay of lipid peroxides in animal tissues by thiobarbituric acid reaction. Anal. Biochem..

[79] Qu K, Chen CP, Halliwell B, Moore PK, Wong PT (2006). Hydrogen sulfide is a mediator of cerebral ischemic damage. Stroke..

[80] Wang L (2004). Modulation of cystathionine beta-synthase level regulates total serum homocysteine in mice. Circ Res..

[81] Kalani A (2014). Nutri-epigenetics ameliorates blood-brain barrier damage and neurodegeneration in hyperhomocysteinemia: role of folic acid. J Mol Neurosci..

[82] Kamat PK (2013). Hydrogen sulfide attenuates neurodegeneration and neurovascular dysfunction induced by intracerebral-administered homocysteine in mice. Neuroscience.

[83] Zhou D, Ren JX, Ryan TM, Higgins NP, Townes TM (2004). Rapid tagging of endogenous mouse genes by recombineering and ES cell complementation of tetraploid blastocysts. Nucleic Acids Re..

[84] Tyagi N (2010). Hydrogen sulfide mitigates matrix metalloproteinase-9 activity and neurovascular permeability in hyperhomocysteinemic mice. Neurochem Int..

[85] Muradashvili N, Benton RL, Saatman KE, Tyagi SC, Lominadze D (2015). Ablation of matrix metalloproteinase-9 gene decreases cerebrovascular permeability and fibrinogen deposition post traumatic brain injury in mice. Metab Brain Dis.

[86] Myojin K (2007). Visualization of intracerebral arteries by synchrotron radiation microangiography. AJNR Am J Neuroradiol.

[87] Givvimani S, Sen U, Tyagi N, Munjal C, Tyagi SC (2011). X-ray imaging of differential vascular density in MMP-9−/−, PAR-1−/+, hyperhomocysteinemic (CBS−/+) and diabetic (Ins2−/+) mice. Arch PhysiolBiochem.

[88] Pushpakumar SB, Kundu S, Metreveli N, Tyagi SC, Sen U (2013). Matrix Metalloproteinase Inhibition Mitigates Renovascular Remodeling in Salt-Sensitive Hypertension. Physiol Rep.

[89] Nooteboom A, Bleichrodt RP, Hendriks T (2006). Modulation of endothelial monolayer permeability induced by plasma obtained from lipopolysaccharide-stimulated whole blood. ClinExpImmunol.

[90] Muradashvili N (2012). Fibrinogen-induced increased pial venular permeability in mice. J Cereb Blood Flow Metab.

