# Supplementary material for: Exercise Mitigates Alcohol Induced Endoplasmic Reticulum Stress Mediated Cognitive Impairment through ATF6-Herp Signaling
Source: Sci Rep. 2018 Mar 26;8:5158. doi: 10.1038/s41598-018-23568-z (PMC5980102; doi:10.1038/s41598-018-23568-z)
Supplement: Supplementary file 1 — Supplementary information [file 41598_2018_23568_MOESM1_ESM.pdf]

Supplementary information

(Full length blots/gels for main figures in the paper)

Exercise Mitigates Alcohol Induced Endoplasmic Reticulum Stress Mediated Cognitive Impairment through ATF6-Herp Signaling

Akash K. George[1,], Jyotirmaya Behera[1,], Kimberly E. Kelly[1], Nandan K. Mondal[1], Kennedy P. Richardson[1], Neetu Tyagi[1,*]

[1]*Department of Physiology, University of Louisville School of medicine, Louisville, KY 40202*

Full-length blots for Figure 3

Fig.3c: CBS

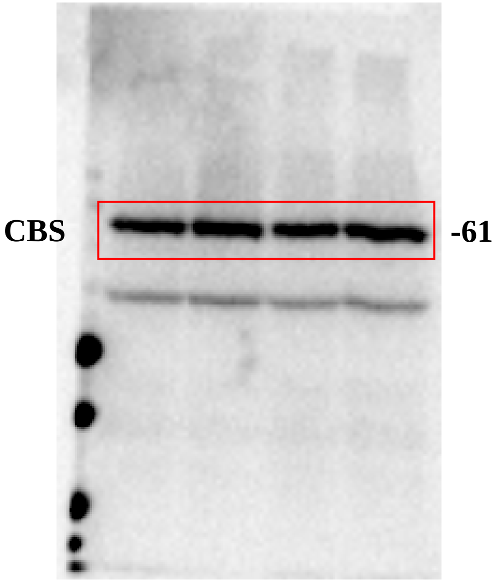

Fig.3c: CSE

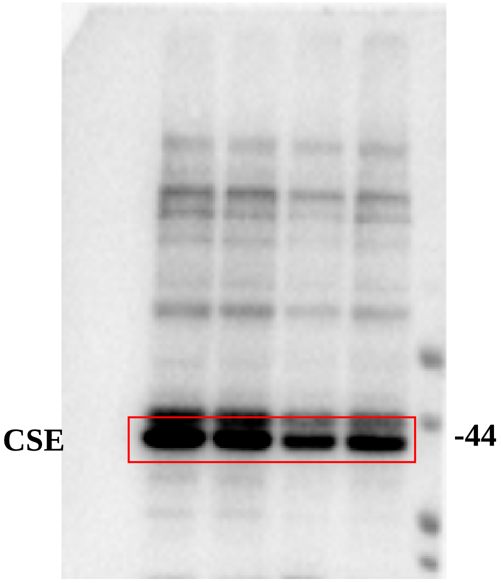

Fig.3c: GAPDH

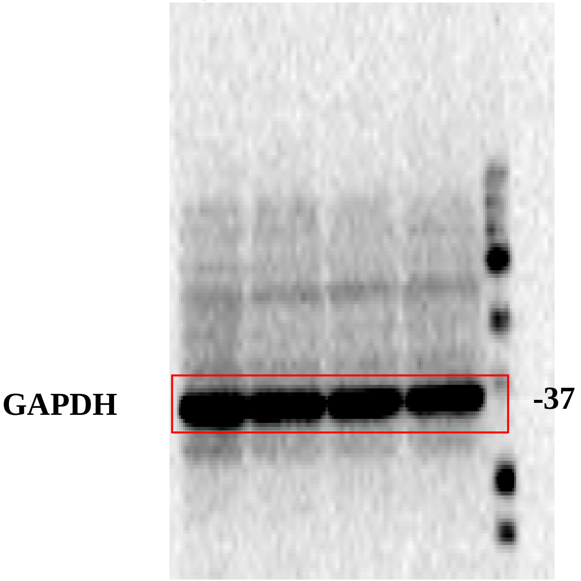

Supplementary Fig. S1: Uncropped gels/blots for main Figs. 3c. Boxed regions are used in the main figures. For some data, transferred membranes were horizontally cut for applying multiple antibodies. Lanes for unrelated samples are deleted. Lanes from left to right in red box: CT, EX, AL, AL+EX.

Full-length blots for Figure 3

Fig.3e: MTHFR

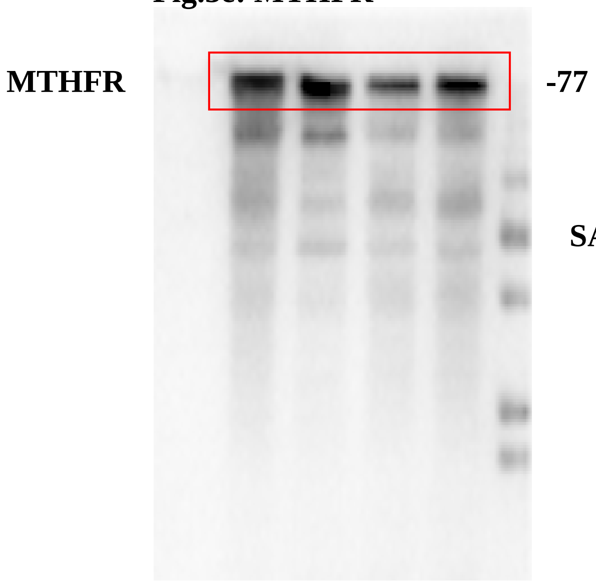

Fig.3e: SAHH

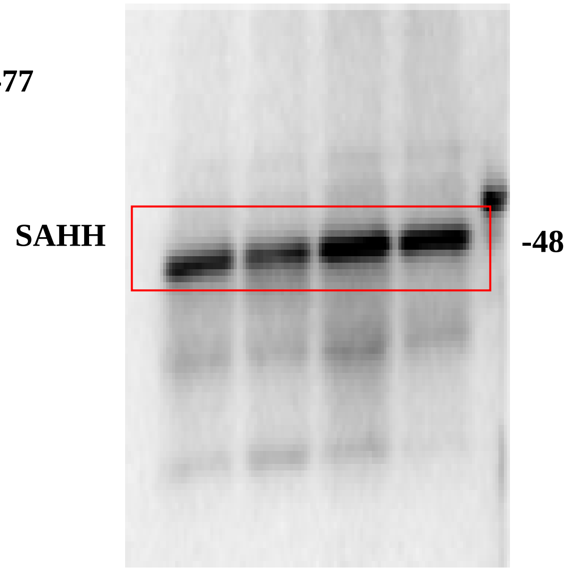

Fig.3e: Hcy

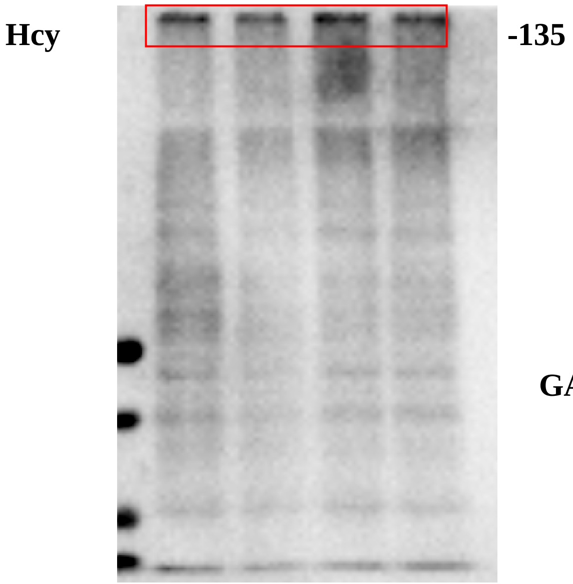

Fig.3e: GAPDH

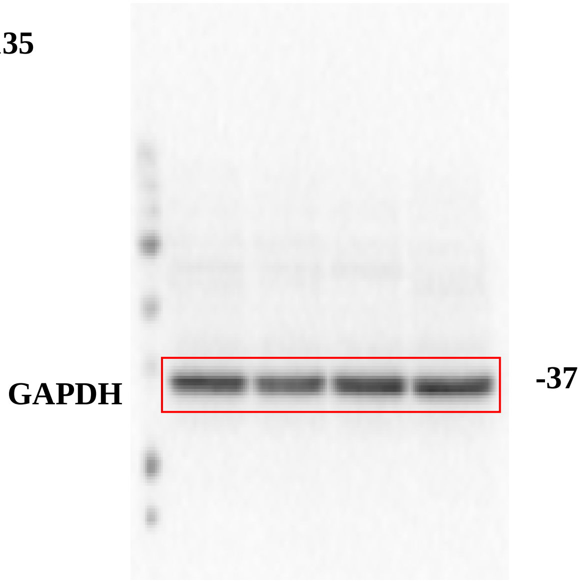

Supplementary Fig. S2: Uncropped gels/blots for main Figs. 3c. Boxed regions are used in the main figures. For some data, transferred membranes were horizontally cut for applying multiple antibodies. Lanes for unrelated samples are deleted. Lanes from left to right in red box: CT, EX, AL, AL+EX.

Full-length blots for Figure 4

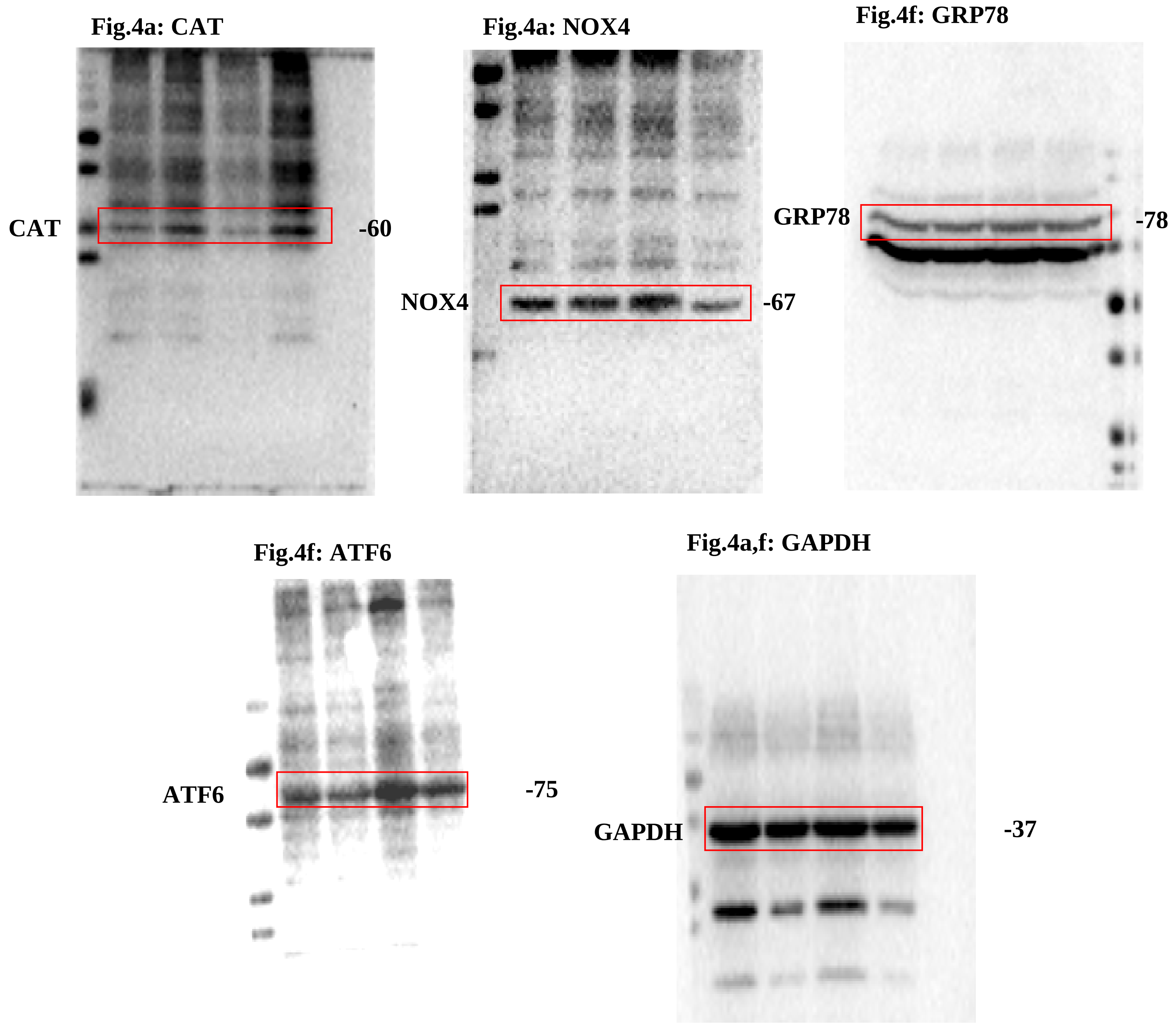

Supplementary Fig. S3: Uncropped gels/blots for main Figs. 4a,f. Boxed regions are used in the main figures. For some data, transferred membranes were horizontally cut for applying multiple antibodies. Lanes for unrelated samples are deleted. Lanes from left to right in red box: CT, EX, AL, AL+EX.

Full-length blots for Figure 5

Fig.5a: DNMT1

DNMT
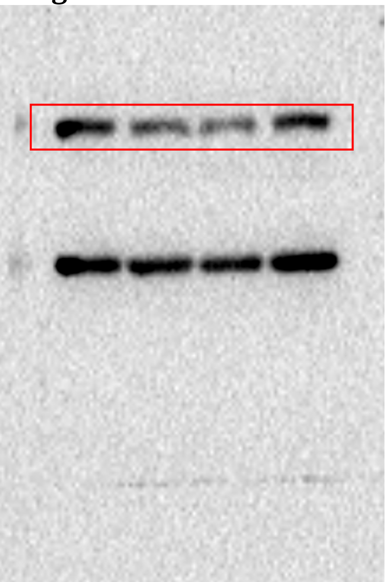
-183

Fig.5a: DNMT3a

DNMT3a
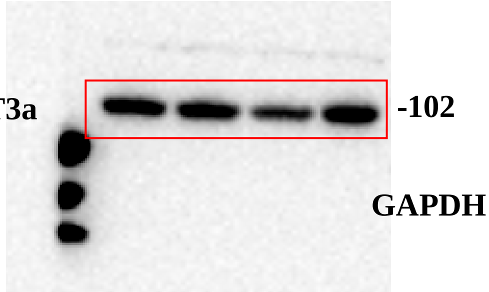
-102

Fig.5a: GAPDH

GAPDH
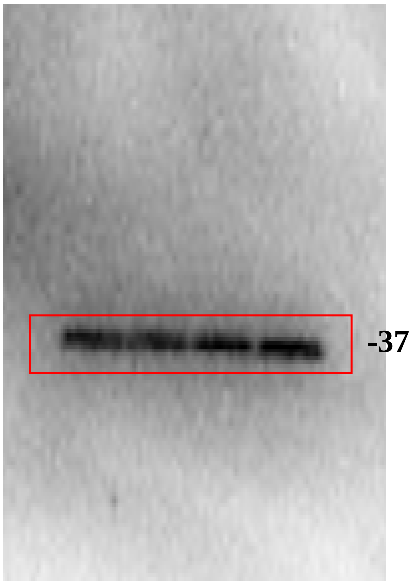
-37

Fig.5e: Herp chip

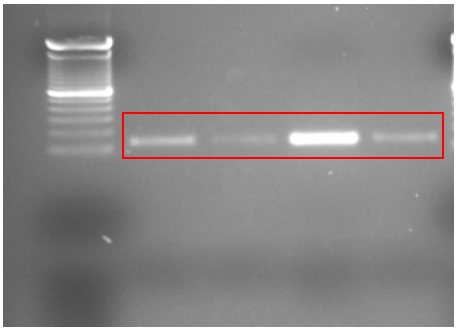

Fig.5e: Input DNA

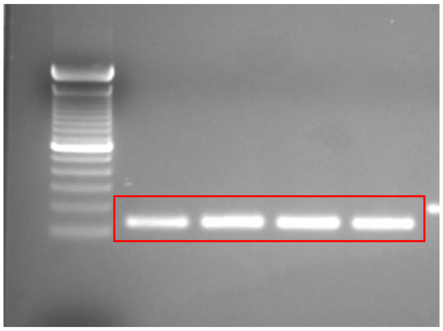

Supplementary Fig. S4: Uncropped gels/blots for main Figs. 5a,e. Boxed regions are used in the main figures. For some data, transferred membranes were horizontally cut for applying multiple antibodies. Lanes for unrelated samples are deleted. Lanes from left to right in red box: CT, EX, AL, AL+EX.

Full-length blots for Figure 5

Fig.5g: Herp

Herp 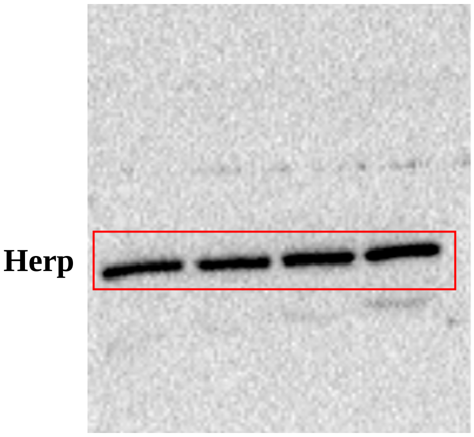 **-44**

Fig.5g: GAPDH

GAPDH 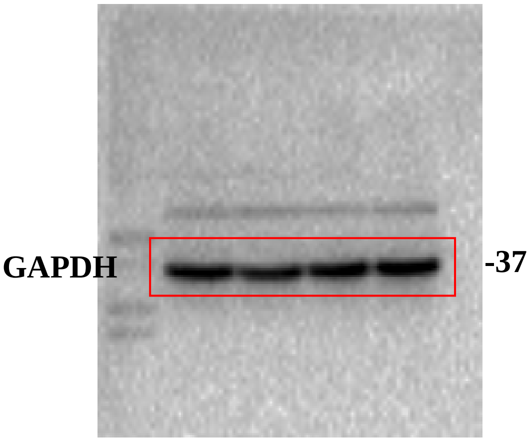 **-37**

Supplementary Fig. S5: Uncropped gels/blots for main Figs. 5g. Boxed regions are used in the main figures. For some data, transferred membranes were horizontally cut for applying multiple antibodies. Lanes for unrelated samples are deleted. Lanes from left to right in red box: CT, EX, AL, AL+EX.

Full-length blots for Figure 6

Fig.6a: ZO1

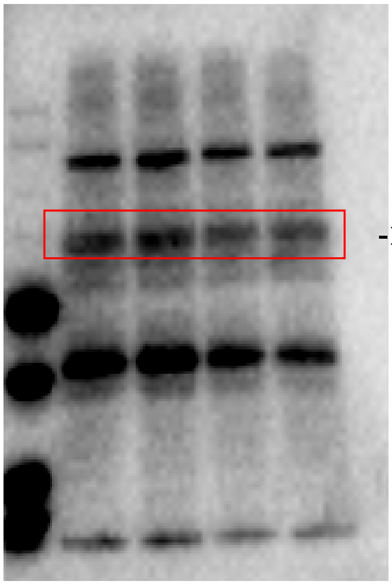

Fig.6a: Claudin-5

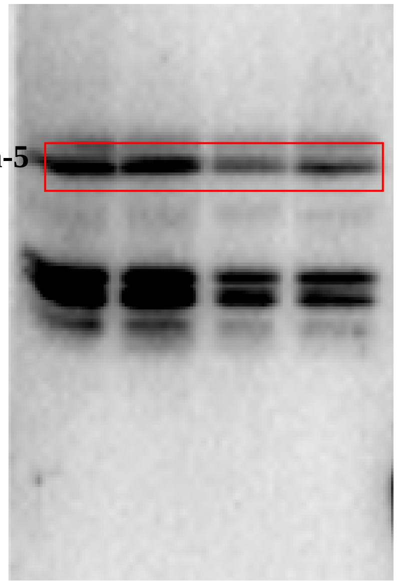

Fig.6a: GAPDH

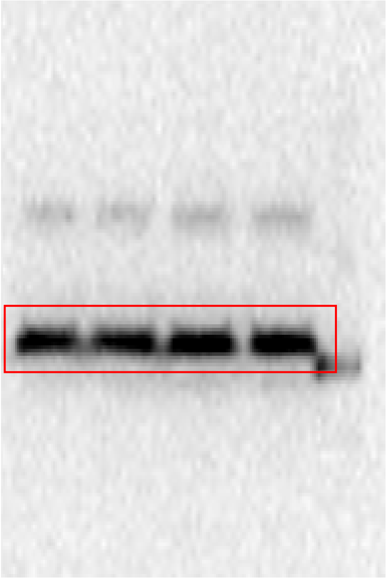

Supplementary Fig. S6: Uncropped gels/blots for main Figs. 6a. Boxed regions are used in the main figures. For some data, transferred membranes were horizontally cut for applying multiple antibodies. Lanes for unrelated samples are deleted. Lanes from left to right in red box: CT, EX, AL, AL+EX.

Full-length blots for Figure 6

Fig.6i: VE-Cadherin

VE-Cadherin

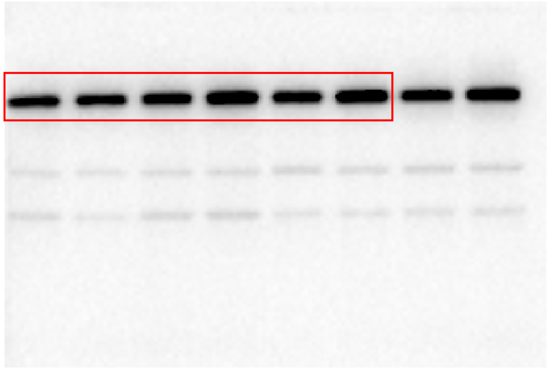

-110

Fig.6i: Claudin-5

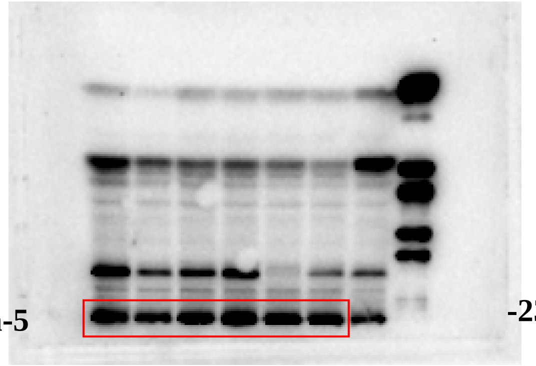

Claudin-5

-23

Fig.6i: ZO2

ZO2

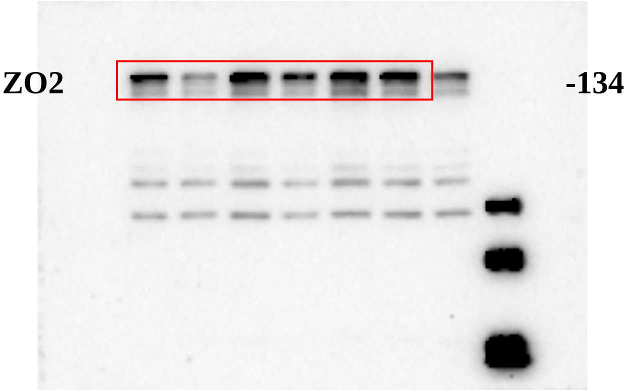

-134

Fig.6i: GAPDH

GAPDH

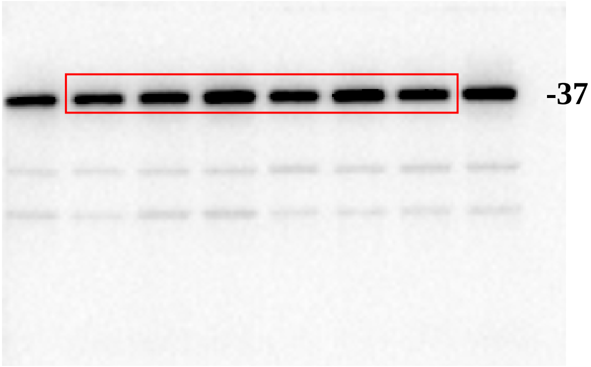

-37

Supplementary Fig. S7: Uncropped gels/blots for main Figs. 6i. Boxed regions are used in the main figures. For some data, transferred membranes were horizontally cut for applying multiple antibodies. Lanes for unrelated samples are deleted. Lanes from left to right in red box: CT, AL, H_2S, AL+H_2S, DTT, AL+DTT.

Full-length blots for Figure 7

Fig.7a: nNOS

nNOS

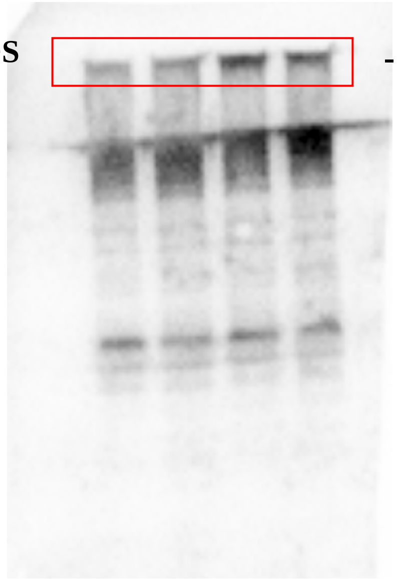

-160

Fig.7a: NSE

NSE

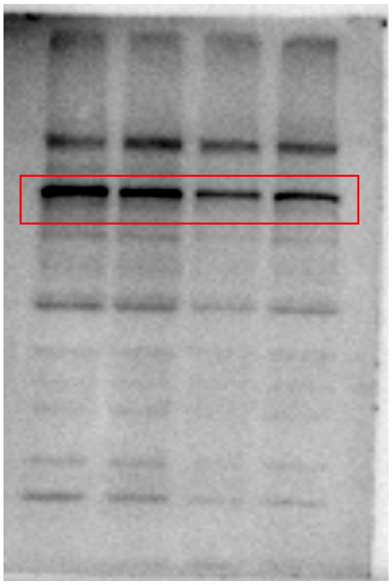

-47

Fig.7a: GAPDH

GAPDH

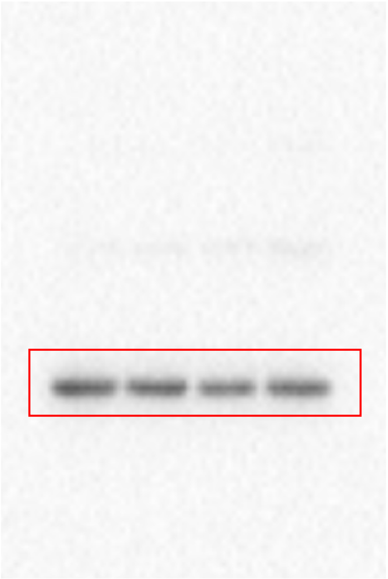

-37

Supplementary Fig. S8: Uncropped gels/blots for main Figs. 7a. Boxed regions are used in the main figures. For some data, transferred membranes were horizontally cut for applying multiple antibodies. Lanes for unrelated samples are deleted. Lanes from left to right in red box: CT, EX, AL, AL+EX.
